# Ligand Sulfur Oxidation State Progressively Alters Galectin-3-Ligand Complex Conformations To Induce Affinity-Influencing Hydrogen Bonds

**DOI:** 10.1021/acs.jmedchem.3c01223

**Published:** 2023-10-25

**Authors:** Mukul Mahanti, Kumar Bhaskar Pal, Rohit Kumar, Markus Schulze, Hakon Leffler, Derek T. Logan, Ulf J. Nilsson

**Affiliations:** † Department of Chemistry, Lund University, Box 124, SE-221 00 Lund, Sweden; ‡ Division of Biochemistry & Structural Biology, Centre for Molecular Protein Science, Department of Chemistry, Lund University, Box 124, SE-221 00 Lund, Sweden; § Department of Laboratory Medicine, Section MIG, Lund University, BMC-C1228b Klinikgatan 28, 221 84 Lund, Sweden

## Abstract

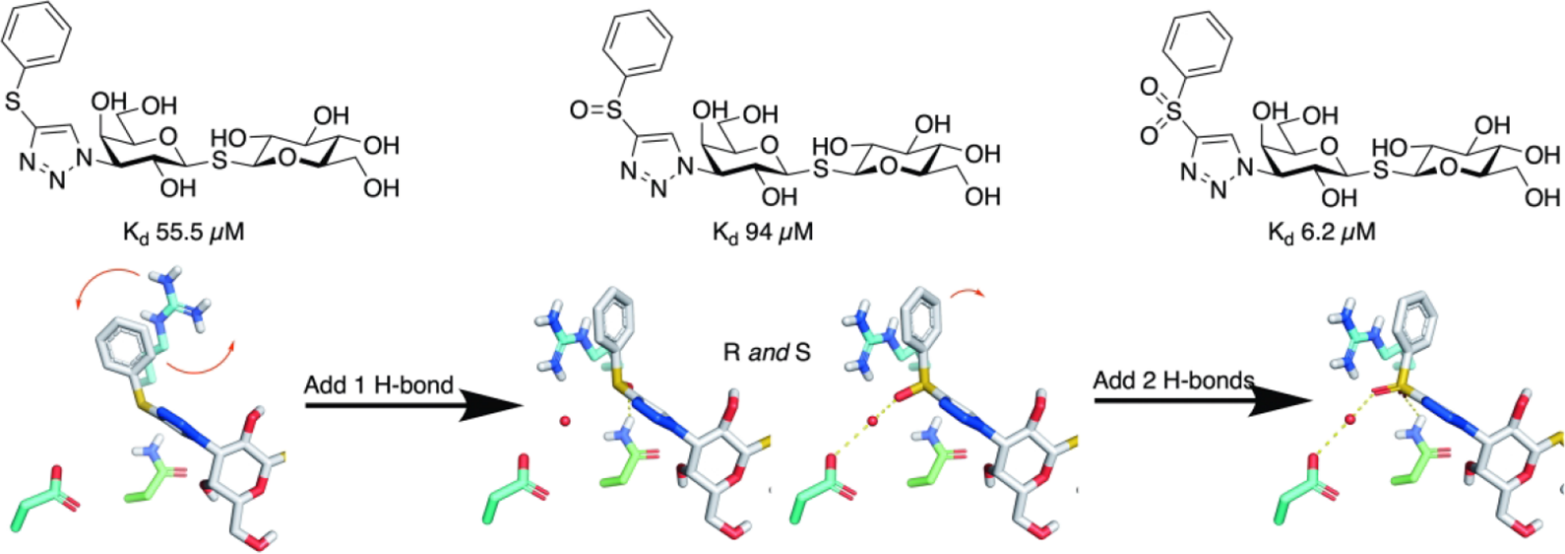

Galectins play biological roles in immune regulation and tumor progression. Ligands with high affinity for the shallow, hydrophilic galectin-3 ligand binding site rely primarily on a galactose core with appended aryltriazole moieties, making hydrophobic interactions and π-stacking. We designed and synthesized phenyl sulfone, sulfoxide, and sulfide-triazolyl thiogalactoside derivatives to create affinity-enhancing hydrogen bonds, hydrophobic and π-interactions. Crystal structures and thermodynamic analyses revealed that the sulfoxide and sulfone ligands form hydrogen bonds while retaining π-interactions, resulting in improved affinities and unique binding poses. The sulfoxide, bearing one hydrogen bond acceptor, leads to an affinity decrease compared to the sulfide, whereas the corresponding sulfone forms three hydrogen bonds, two directly with Asn and Arg side chains and one water-mediated to an Asp side chain, respectively, which alters the complex structure and increases affinity. These findings highlight that the sulfur oxidation state influences both the interaction thermodynamics and structure.

## Introduction

Structural and thermodynamic analyses are of key importance for understanding target protein–ligand interactions and, thus, for facilitating drug discovery. Identifying and characterizing potential sites for H-bonds, π interactions, ionic, and other polar interactions between protein and ligand^1−3^ is at the center of this process. A common strategy to improve affinity between a ligand and a protein is to introduce hydrophobic, typically aromatic, structural moieties in a lead structure to interact with, e.g., hydrophobic, aromatic, and/or cationic parts of the protein site. However, this approach may lead to overly hydrophobic molecules with potentially less favorable absorption, distribution, metabolism, and excretion (ADME) properties and poor solubility. Therefore, introducing polar interactions during lead optimization can be advantageous to enhance the affinity.

An ideal model for studies of ligand–protein interactions is galectin-3, which belongs to a family of lectins having a natural affinity for β-D-galactopyranosides.^4^ The choice of galectin-3 is advantageous for studies of protein–ligand interactions, as it is easily expressed, stable in solution to high concentrations, amenable to nuclear magnetic resonance (NMR) spectroscopic experiments, and crystallizes and diffracts to high resolution with ligands complexed. Hence, a large number of structural elucidations, thermodynamic studies, and molecular dynamics (MD) simulations have been done with galectin-3 as a model protein.^2,3,5−8^ For example, the flexible side chain Arg144 has been demonstrated to play an important role in ligand binding.^9,10^ In the presence of inhibitors equipped with aryl moieties at C3 of the galactose moiety, Arg144 rearranges from its location on the surface of the galectin-3 carbohydrate recognition domain (CRD), in a water-mediated salt bridge to Asp148, to a new position in which it creates a binding pocket partly mediated by cation−π interactions with the ligand aryl group.^11^ More recently, we have carried out several systematic studies of the interactions of arene moieties in synthetic ligands with Arg144, in particular how the choice of different aromatic groups or substituents influences the affinity.^3,6,9,12^ Recent studies of solvation and fluorination effects on the Arg144-arene interactions with a series of phenyltriazole-derivatized galactosides^6^ raised the question of whether the conformation of Arg144, and consequently the ligand affinity, could be further influenced by introducing hydrogen bonding functionalities in the ligand structure. The arene–Arg144 interaction has been exploited in the development of topical^13,14^ and orally^8,15^ administered drugs currently in clinical trials. Herein, we present the synthesis of a sulfide-sulfoxide-sulfone series of galectin-3 ligands together with evaluations of their affinity and binding thermodynamics combined with high-resolution structural analysis in order to understand hydrogen-bond-assisted aryl−arginine face-to-face stacking in galectin-3-ligand complexes. The results suggest that arene–Arg144 and hydrogen bond interactions can be tuned by changing the ligand sulfur oxidation state, leading to affinity enhancement and galectin-3 ligand site reorganization to novel ligand-bound conformations.

## Results and Discussion

### Synthesis and Affinity Evaluations

**Scheme 1:**
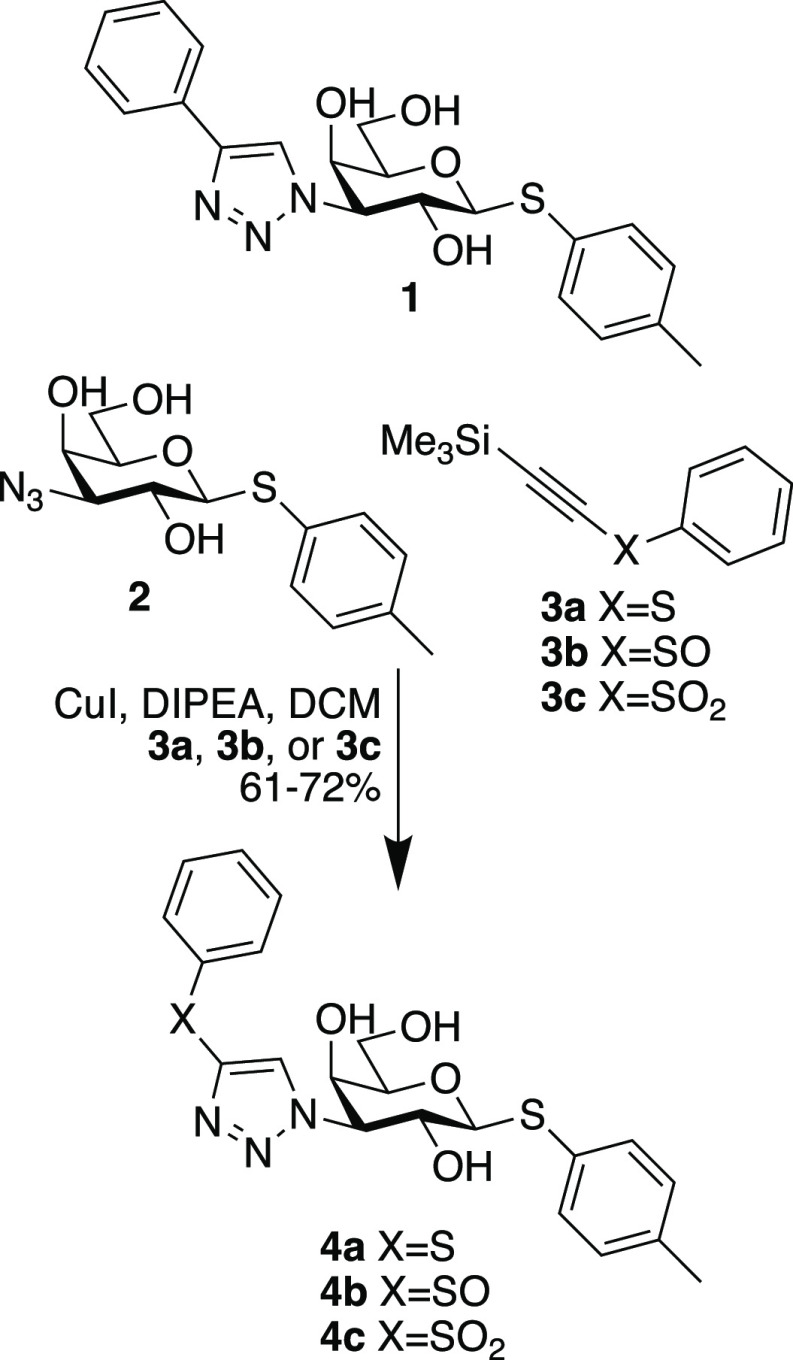
Reference Compound **1** Structure and the Synthesis of Compounds **4a**, **4b**, and **4c**

Initially, a series of analogous galactosides derivatized at the pyranose C3 position with phenylsulfide **4a**, sulfoxide **4b**, and sulfone **4c** at the triazole C4 position was synthesized (Scheme 1) and evaluated and compared to the phenyltriazole derivative **1** for galectin-3 interaction characteristics (Table 1). Synthesis of compounds **4a**–**4c** was done from the known *p*-methylphenyl 3-azido-3-deoxy-β-D-thiogalactopyranoside **2**^6^ via CuI-mediated 1,3-dipolar cycloaddition with (trimethylsilylethynylsulfanyl)-benzene **3a**, trimethylsilylethynyl phenyl sulfoxide **3b**, and trimethylsilylethynyl phenyl sulfone **3c**, respectively, and with spontaneous in situ desilylation (Scheme 1). Evaluation of the galectin-3 affinities of these ligands in a competitive fluorescence polarization assay (Table 1) showed that the sulfide **4a** bound more weakly to galectin-3 than the reference,^1^ with a *K*_d_ of 260 μM compared to 88 μM for the reference. The racemic sulfoxide **4b** had an even weaker affinity (*K*_d_ 600 μM), while the sulfone regained and marginally improved affinity over that of **1,** with *K*_d_ of 79 μM. The phenyltriazole moiety has been shown to interact with Arg144 in galectin-3; thus, to gain an understanding of why the phenylsulfone **4c** regained affinity, it was evaluated for affinity against the galectin-3 R144S^13,16^ and R144K^13^ mutants in the fluorescence polarization assay. A critical role for the Arg144 side chain was revealed, as **4c** bound more weakly to both mutants, with *K*_d_ values of 600 μM for R144S and 250 μM for R144K. The somewhat higher affinity of R144K than that of R144S may indicate that a cationic and/or larger side chain forms beneficial interactions with the phenyl sulfone moiety of **4c**.

**Table 1:** *K*_d_ Values (μM) for Compounds **1** and **4a**–**4c** against Galectin-3 Wild-Type, R144S, and R144K

compound	*K*_d_ (μM)	*K*_d_ (R144S)	*K*_d_ (R144K)
**1**^8^	88	NT^a^	NT
**4a**	260 ± 28	NT	NT
**4b**	> 600^b^	NT	NT
**4c**	79 ± 8	600 ± 80	250 ± 6

a NT, not tested.

b Weak inhibition at the highest tested concentration of **4b** (2 mM) did not allow for accurate determination of the *K*_d_ value.

**Scheme 2:**
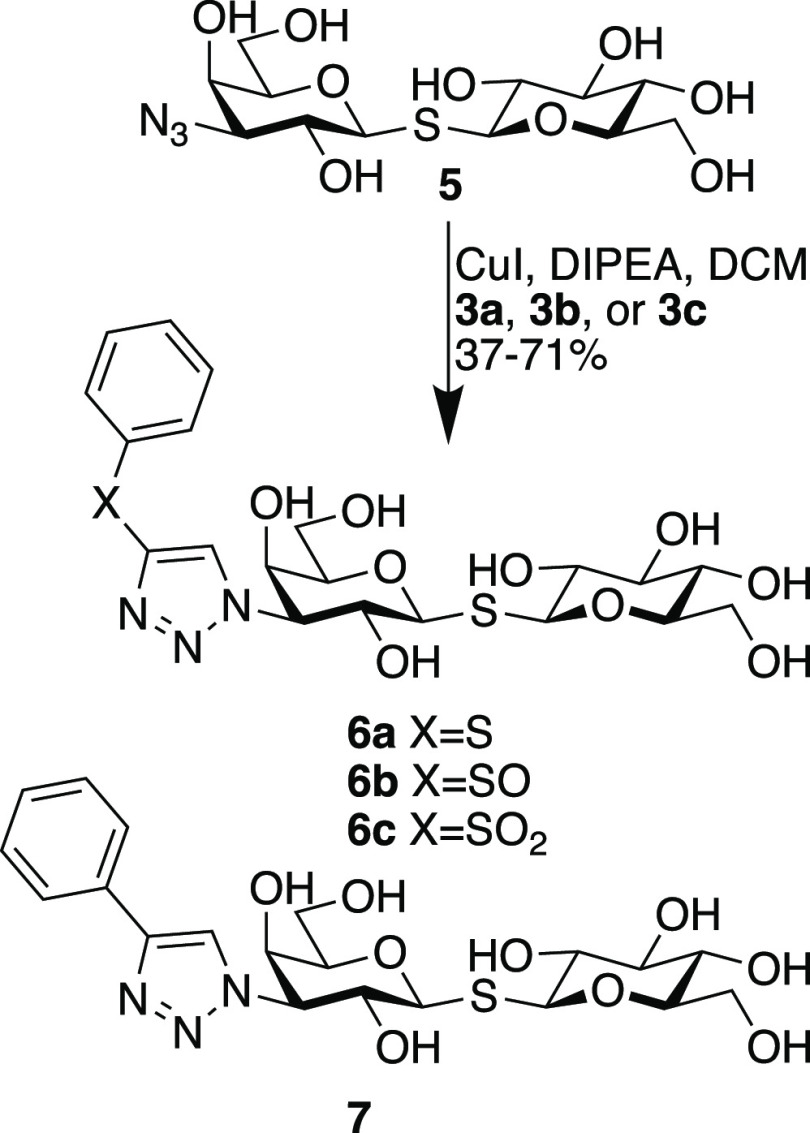
Synthesis of the Thiogalactoglucosides **6a**, **6b**, and **6c** and the Structure of the Reference Phenyltriazole **7**

Initial attempts at crystallographic structural analyses of galectin-3C (the C-terminal domain of galectin-3) with **4a**, **4b**, and **4c** failed due to insufficient solubility of all compounds. Hence, we adopted a strategy to increase the ligand solubility by replacing the hydrophobic tolyl aglycon with a glucose residue. The known thiodiglycoside **5**^6^ was treated with **3a** and **3b** and **3c** in the presence of DIPEA and CuI to afford the corresponding disaccharides **6a**, **6b**, and **6c**, respectively (Scheme 2). Affinity evaluations revealed higher affinities of **6a**–**6c** for galectin-3 but with the same affinity trends among the three ligands and toward the reference phenyltriazole **7**^3^ (Table 2).

**Table 2:** *K*_d_ Values (μM) for Compounds **6a**–**6c** Binding to Galectin-3

compound	*K*_d_ (μM)
**6a**	19 ± 2.6
**6b**	55.5 ± 5.3
**6c**	8.8 ± 0.62
**7**	4.5^17^

### Structural Analysis of Galectin-3C in Complex with **6a**, **6b**, and **6c**

**Figure 1:**
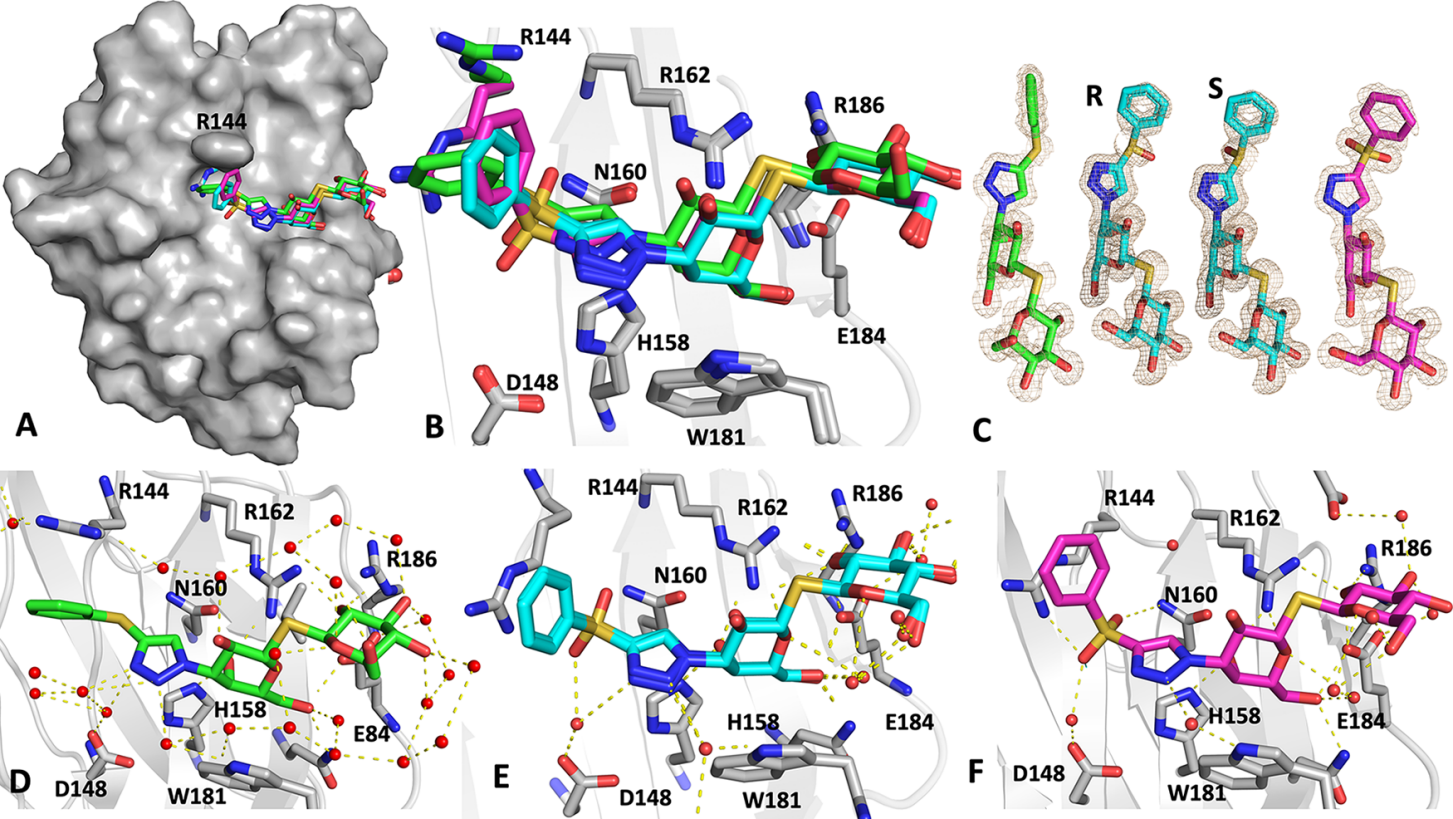
(A) Surface view of the galectin-3C complexes with compounds **6a, 6b**, and **6c.** Arg144 is labeled. Compounds are shown in stick representation. Compound **6a** (PDB ID 8PBF) is shown with carbon atoms colored green, **6b** (8PF9) cyan, and **6c** (8PFF) magenta, respectively. The same color scheme is used in all subsequent panels. (B) Details of the binding mode for the compounds showing all of the key residues in galectin-3. The carbon atoms of the side chain of Arg144 are colored according to the respective ligand to show its two conformational states. The conformation for **6b** is not visible as it is identical to and thus masked by that of **6c**. Other side chains do not vary in conformation between the complexes. (C) 2 | *F*_o_| – | *F*_c_| electron density for the compounds. The R- and S-enantiomers of **6b** are shown separately in the same map. The maps are contoured at a level of 1σ above the mean. (D–F) Individual binding modes and polar contacts of **6a**–**6c**. The ligands are colored as in (C). Panel E shows both enantiomers of **6b**, which are identical except at the sulfur atom; therefore, the interactions of both enantiomers are shown. The differences between these are visualized in Figure 3.

High-resolution X-ray structures of **6a**, **6b**, and **6c** in complex with galectin-3C were obtained at 1.08–1.14 Å resolution (Table 3). In all three complexes, the protein atoms superimpose very well, and there are no significant conformational differences in the protein in the vicinity of the binding site apart from Arg144 (Figure 1). The binding modes of the galactose and glucose moieties are identical to previously reported structures^3,6^ (Figure 1). However, the C3 substituents provide two novel binding modes with Arg144 that depend on the oxidation state of the sulfur atom, neither of which has previously been reported for galectin-3 inhibitors. One face of the phenyl groups of the sulfoxide **6b** and sulfone **6c** is directed toward the solution, resulting in some flexibility, which is reflected by slightly poorer electron density for this moiety compared to the rest of the ligand, especially for the phenyl group of the sulfoxide **6b** (Figure 1C).

**Table 3:** Data Collection Statistics and Model Quality for Crystal Structures of Galectin-3C in Complex with **6a**–**6c**^a^

	**6a**	**6b**	**6c**
PDB code	8PBF	8PF9	8PFF
beamline	BioMAX	BioMAX	BioMAX
wavelength [Å]	0.6357	0.7749	0.7749
unit cell (Å)	*a* = 36.5	*a* = 34.3	*a* = 34.0
	*b* = 57.0	*b* = 57.8	*b* = 57.8
	*c* = 61.9	*c* = 61.9	*c* = 61.6
space group	*P*2_1_2_1_2_1_	*P*2_1_2_1_2_1_	*P*2_1_2_1_2_1_
resolution range [Å]	22.46–1.14 (1.18–1.14)	26.59–1.09 (1.16–1.09)	27.17–1.08 (1.12–1.08)
completeness [%]	98.8 (89.6)	99.9 (100.0)	97.6 (75.8)
total reflections	309,813 (26,930)	434,271 (64,759)	644,446 (44,732)
unique reflections	46,849 (4238)	51,101 (7876)	51,395 (4240)
*CC*_1/2_	0.997 (0.458)	0.997 (0.471)	0.998 (0.376)
multiplicity	6.6 (6.4)	8.5 (8.2)	12.5 (10.5)
*R*_merge_(I) [%]	0.156 (1.443)	0.111 (1.470)	0.124 (1.622)
mean I/σ(I)	6.75 (0.97)	7.45 (0.93)	8.92 (0.85)
Wilson *B*-factor [Å^2^]	8.73	17.0	11.0
*R*_model_(*F*) [%]	0.150 (0.289)	0.206 (0.325)	0.178 (0.585)
*R*_free_(*F*) [%]	0.179 (0.321)	0.224 (0.375)	0.203 (0.666)
reflections used in refinement	46,766 (4182)	51,058 (2458)	51,027 (3877)
for *R*_free_	2534 (224)	2462 (115)	2466 (179)
average *B*-factors [Å^2^]	protein: 9.9	protein: 15.2	protein: 14.4
	ligand: 11.4	ligand: 22.9	ligand:15.0
	solvent: 24.1	solvent: 30.0	solvent: 25.9
Ramachandran outliers [%]	0.0	0.0	0.00
rotamer outliers [%]	0.79	0.76	0.00
MolProbity clash score	0.87	2.1	3.4
bond length RMSD from ideal [Å]	0.009	0.005	0.021
bond angle RMSD from ideal [°]	1.13	0.87	1.70

a Numbers in parentheses describe the highest-resolution shell.

**Figure 2:**
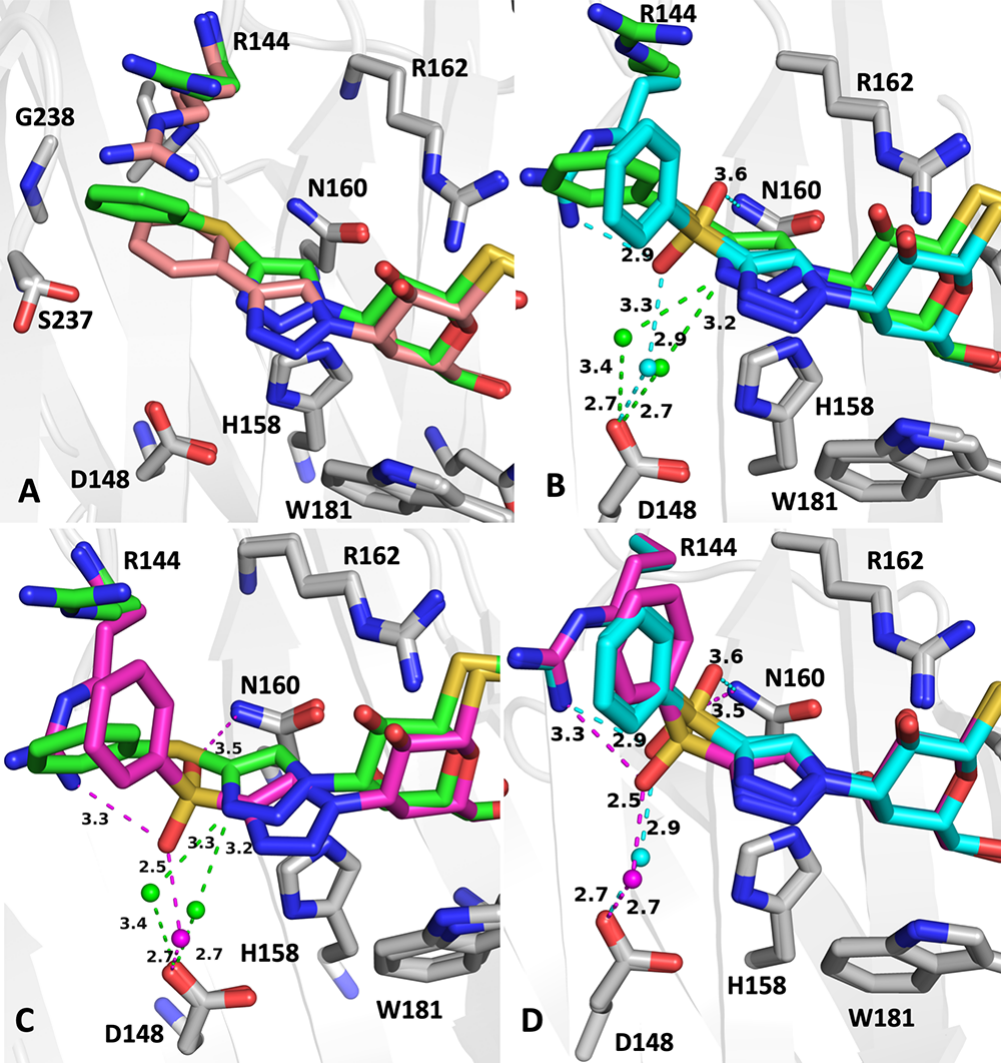
Closeup view of the binding of the compounds near Arg144 in the binding pocket. (A) Comparison of the sulfide **6a** (PDB ID 8PBF, green) and unsubstituted phenyltriazole **7** (pink). (B) Sulfide **6a** (green) and the sulfoxide **6b** (8PF9, both R and S enantiomers shown) (cyan). (C) Sulfide **6a** (green) and the sulfone **6c** (8PFF. magenta). (D) Sulfoxide **6b** (R and S) (cyan) and the sulfone **6c** (magenta). Key polar contacts are shown and color-coded green for **6a**, cyan for **6b** (R and S), and magenta for **6c**. Key water molecules in each structure are shown as small spheres colored the same way as the carbon atoms of the ligand. However, all water molecules are omitted from panel A for clarity. Hydrogen bonds made by the ligands are shown as dashed lines colored as for the C atoms. Hydrogen bond distances are shown in Å.

In the **6a** complex, the introduction of a sulfur atom as a spacer between the triazole and phenyl moieties, compared to the known compound **7**^3^ where the phenyl and triazole rings were directly linked with a bond, results in an inward shift of the entire C3-substituent toward the protein by about 1 Å (Figure 2A). In **6a**, the sulfur atom occupies approximately the same position as the ortho-carbon atom in the unsubstituted phenyl group of the published phenyltriazole **7**. The dihedral angles of the C–S–C bond orient the phenyl group in a new position that causes an upward displacement of the side chain of Arg144 to an orientation different from that of Arg144 in complex with **7**, where Arg144 sandwiches the phenyltriazole moiety between its side chain and that of Ala146. In the **6a** complex, Arg144 is further moved upward to maintain a cation−π interaction with the phenyl group of **6a**, albeit with a poorer overlap between the phenyl group and the guanidinium moiety of Arg144, as the interaction is shifted toward the aliphatic part of the Arg144 side chain (Figure 2A).

**Figure 3:**
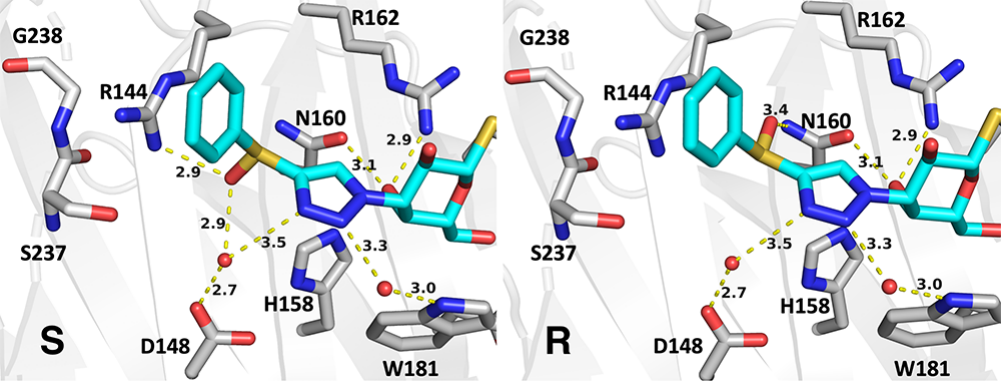
Binding modes of the S and R configurations of **6b** (PDB ID 8PF9). The S-configuration sulfoxide oxygen coordinates Arg144 and a water molecule together with Asp148. The R-configuration sulfoxide oxygen makes a long 3.4 Å contact at a nonideal angle with Asn160. Key distances and polar contacts are shown.

The oxidation of the sulfur atom in **6a** to the sulfoxide **6b** renders the sulfur atom a chiral center and synthesis produces a mixture of R- and S-enantiomers (Figure 2B,D). The electron density for **6b** in the complex is compatible with a mixture of both enantiomers, both of which have an oxygen atom oriented toward the protein. It is difficult to establish the exact ratio of the two enantiomers and they have been modeled with 50% occupancy each. Surprisingly, **6b** also induces a new conformation of Arg144 that has not been seen in any galectin-3C structure to date. In this conformation, Arg144 is sandwiched between the phenyl group of **6b** and the π-system of the amide bond of Ser237-Gly238 (Figure 3). In the previous phenyltriazole ligand series, Ser237-Gly238 represented the inner boundary of the aryl binding pocket that was exploited for fluorine-amide interactions with the main chain atoms of these two residues.^3^ In this novel position, the guanidinium group of Arg144 makes two hydrogen bonds: one to the side chain hydroxyl of Ser237 and the other to the sulfoxide oxygen of S-**6b**. The binding mode of the sulfone **6c** is very similar to that of the sulfoxide **6b** (Figure 2C,D), which is logical since the sulfur atom has tetrahedral geometry in both cases, and **6c** thus mimics the simultaneous binding of both enantiomers of **6b**. The sulfoxide and sulfonyl groups of S-**6b** and **6c,** respectively, together with the triazole of all compounds, coordinate a water molecule that bridges to the Asp148 side chain, but the distances between the water molecules, residues, and the ligand vary (Figure 2C,D). In the case of sulfide **6a**, only the triazole nitrogen binds the water molecule (Figure 1D) at 3.3 Å from the triazole nitrogen and 2.7 Å from Asp148. For sulfoxide **6b**, the water molecule is 2.9 Å from the sulfoxide oxygen of S-**6b** and 2.7 Å from Asp148 (Figures 1E and 2B,C). In **6c**, the water molecule is 2.6 Å from the nearest sulfone oxygen and 2.7 Å from Asp148 (Figure 1F). The introduction of a second oxygen on the sulfur atom in **6c** also pushes the water molecule toward bulk solvent, thus increasing the distance between Asp148 and the water molecule. Furthermore, the sulfoxide oxygen in S-**6b** forms an H-bond with Arg144 and the water molecule, and R-**6b** sulfoxide oxygen is placed near N160, albeit at a less-than-optimal interaction distance and angle.

### Thermodynamic Analysis

**Figure 4:**
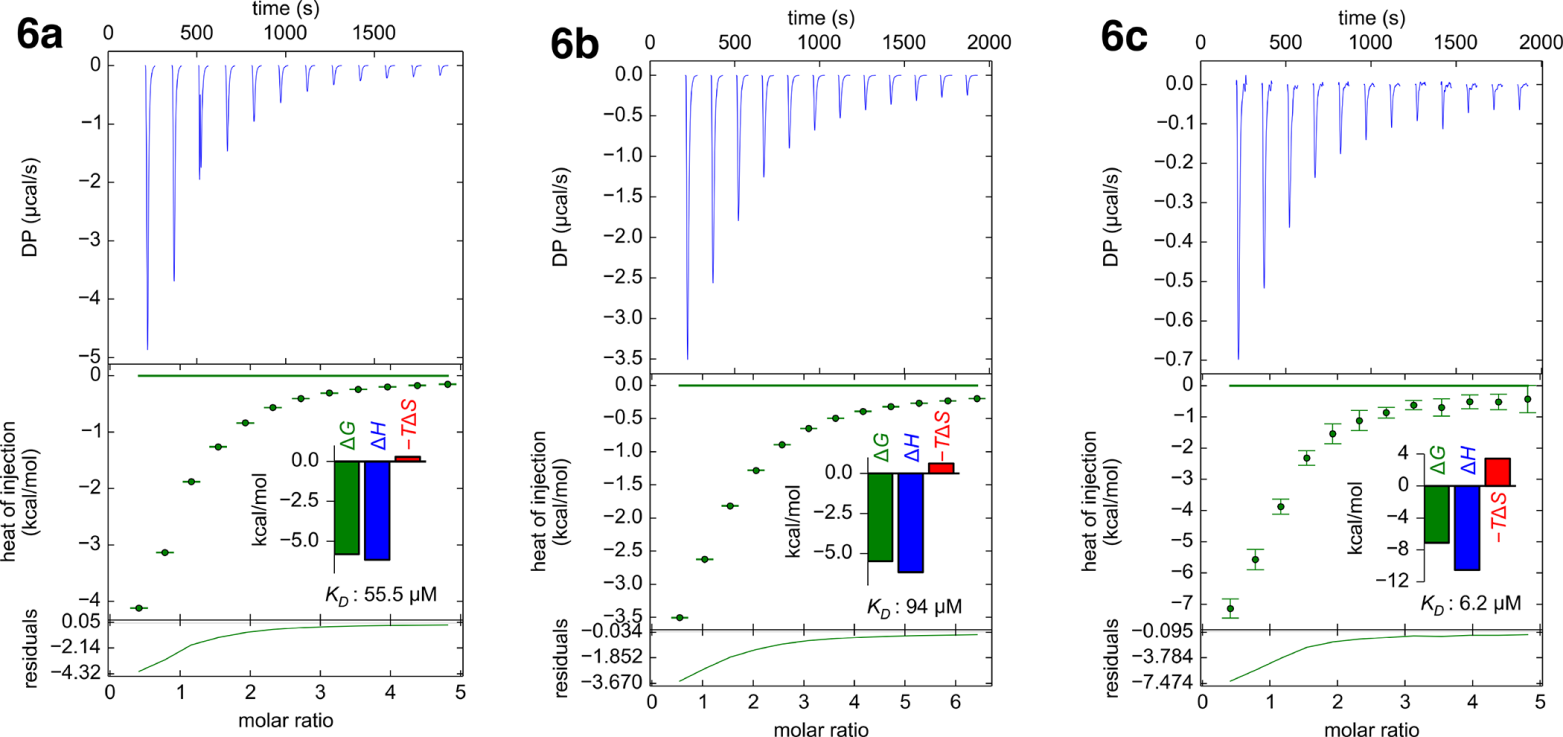
ITC thermograms of galectin-3C titrated into compounds **6a**, **6b**, and **6c**.

Isothermal titration calorimetry (ITC) of **6a**–**6c** with galectin-3C was performed to gain a further understanding of the thermodynamics of binding of **6a**–**6c** to galectin-3. Galectin-3C was titrated into the ITC cells containing **6a**–**6c** (Table 4 and Figure 4). The enthalpies of binding of the sulfide **6a** and sulfoxide **6b** were −6.17 ± 0.15 and −6.15 ± 0.39 kcal/mol, respectively, whereas the TΔ*S* terms were 0.29 and 0.62 kcal/mol, which translates to *K*_d_ values of 55.5 ± 7.89 and 94.2 ± 3.7 μM for **6a** and **6b**, respectively. The higher affinity of **6c** was confirmed by an enthalpic contribution of −10.5 ± 1.5 kcal/mol and *T*Δ*S* of 3.4 kcal/mol, giving *K*_d_ of 6.2 ± 0.18 μM. Hence, the ITC affinities of **6a**–**6c** correlate overall with those obtained with the competitive fluorescence polarization assay. The higher affinity of **6c** is primarily a result of enhanced enthalpy of binding compared to **6a**–**b**, albeit with an accompanying larger entropy penalty, which may be a consequence of added hydrogen bonds to galectin-3C. The sulfoxide **6b** also forms hydrogen bonds to galectin-3C, but the two diastereomers do so with different residues; R-**6b** binds the Asn160 side chain amide, while S-**6b** binds directly to Arg144 and to Asp148 via a water molecule (Figure 3). One may speculate that the two different hydrogen bonding patterns of the two diastereomers of **6b** display different thermodynamic characteristics that may at least partly counteract each other in the ITC analysis of the **6b** mixture. The sulfone **6c** forms all hydrogen bonds to Asn160, Arg144, and water-mediated to Asp148, which is likely reflected in the significantly greater enthalpy of binding compared to **6a** and **6b** but also in the greater entropic penalty.

**Table 4:** ITC Data for Galectin-3C Titrated into Compounds **6a**, **6b**, and **6c**

compound	Δ*H* (kcal/mol)	*T*Δ*S* (kcal/mol)	Δ*G* (kcal/mol)	*K*_d_ (ITC)	*K*_d_ (FP)
**6a**	– 6.15 ± 0.15	– 0.29 ± 0.17	5.86 ± 0.078	55.5 ± 7.89	19 ± 2.6
**6b**	– 6.17 ± 0.39	– 0.62 ± 0.46	– 5.55 ± 0.023	94.0 ± 3.73	55.5 ± 5.3
**6c**	– 10.5 ± 1.5	– 3.4 ± 1.	– 7.11 ± 0.15	6.2 ± 0.18	8.8 ± 0.62

## Conclusions

Sulfide, sulfoxide, and sulfone-derivatized galectin-3-binding ligands **6a**–**c** were synthesized, and their binding thermodynamics and complex structures were analyzed. According to X-ray structural analysis, a sulfoxide diastereomeric mixture of the disaccharide ligand **6b** formed hydrogen bonds to galectin-3C not seen for the parent sulfide **6a**, but this resulted in no significant influence on the thermodynamics of binding, and the sulfoxide displayed a somewhat weaker affinity. A possible explanation may be that one of the **6b** diastereomers binds significantly weaker than the other, resulting in a weak affinity of the **6b** diastereomeric mixture. On the other hand, sulfone **6c** formed two additional hydrogen bonds to galectin-3 compared to sulfide **6a**, which translated to a significantly more favorable enthalpy of binding and higher affinity. Simultaneously to the stepwise addition of ligand-galectin-3C hydrogen bonds in the sulfide-sulfoxide-sulfone series **6a**–**6c**, a sulfur-linked ligand phenyl shifted its position in the complex with galectin-3 which led to an accompanying gradual movement of the Arg144 side chain position. These results emphasize the significance of the sulfur oxidation state in optimizing the interaction and structure of galectin-3 ligands, opening new avenues for developing pharmaceuticals targeting galectins.

## Experimental Section

### Chemistry

All reactions were carried out in oven-dried glassware. All solvents and reagents were mainly purchased from Sigma-Aldrich or Fluka and used without further purification or synthesized via literature protocols. Thin-layer chromatography (TLC) analysis was performed on precoated Merck silica gel 60 F_254_ plates using UV light and charring solution (10 mL conc. H_2_SO_4_/ 90 mL EtOH). Flash column chromatography was done on SiO_2_ purchased from Aldrich (technical grade, 60 Å pore size, 230–400 mesh, 40–63 μm). All NMR spectra were recorded with a Bruker DRX 400 MHz spectrometer (400 MHz for ^1^H, 100 MHz for ^13^C, ESI) at ambient temperature using CDCl_3_ and CD_3_OD as solvents. Chemical shifts are given in parts per million relative to the residual solvent peak (^1^H NMR: CDCl_3_δ 7.26; CD_3_OD 3.31; ^13^C NMR: CDCl_3_ δ 77.16; CD_3_OD δ49.00) with multiplicity (b = broad, s = singlet, d = doublet, t = triplet, q = quartet, quin = quintet, hept = heptet, m = multiplet, app = apparent), coupling constants (in Hz), and integration. High-resolution mass analyses were obtained using a Micromass quadrupole time-of-flight (Q-TOF) mass spectrometer. Analytical data are given if the compound is novel or not fully characterized in the literature. Purity analysis was performed using ultraperformance liquid chromatography/mass spectrometry (UPLC/MS) with UV/vis detection on a Waters Acquity UPLC + Waters XEVO-G2 system using a Waters Acquity CSH C18, 1.7 um, 2.1 × 100 mm column. Samples were run using a gradient with water (0.1% formic acid) and acetonitrile using a flow rate of 0.50 mL/min and a column temperature of 60 °C. Gradient parameters: 0–0.7 min: 40% acetonitrile, 0.7–10.0 min: 40–99% acetonitrile, 10.0–11.0 min 99% acetonitrile, 11.0–11.1 min 99–40% acetonitrile, 11.1–13 min 40% acetonitrile, 3 or 6 μL injection, and detection 190–300 nm. Analytical purities were determined at 254 nm detector wavelength. MS parameters: cap voltage 3.0 kV, cone voltage 40 kV, Ext 4, source temp 120 °C, desolvation temp 500 °C, cone gas 50, desolvation gas 800, centroid resolution mode, *m*/*z* interval 50–1200, lockspray. calibration: Leu-enkephalin *m*/*z* 556.2771, 0.25 s every 30 s, average 3. All final compounds were purified using preparative high-performance liquid chromatography (HPLC) on an Agilent 1260 Infinity system with a Symmetry Prep C18 5 μM 19 × 100 mm column using a gradient (water with 0.1% formic acid and acetonitrile), 0–20 min 10–100% acetonitrile and 20–23 min 100% acetonitrile. Monitoring and collection based on UV/vis absorbance at 210 and 254 nm. Purities for compounds **4a**, **4b**, and **4c** were >95%, while compounds **6a**, **6b**, and **6c** were of lower purity (see below).

#### [2-(Trimethylsilyl)ethynyl)-sulfinyl]-benzene **3b**

To a solution of **3a**(17) (200 mg, 1.5 mmol) in dry DCM (10 mL), *m*-chloroperoxybenzoic acid was added (mCPBA; 283 mg, 1.1 mmol), and the reaction was stirred at the room temperature for 3 h after TLC confirmed the full consumption of **3a**. The reaction was quenched with a Na_2_S_2_O_3_ solution (10 mL) and then washed with Na_2_S_2_O_3_ (saturated, 25 mL), NaHCO_3_ (saturated, 25 mL), and NaCl (saturated, 25 mL) solutions. The organic layer was dried (Na_2_SO_4_), filtered, and concentrated to afford **3b** (180 mg, 81% yield), which was used directly without further purification. ^1^H NMR (CDCl_3_, 400 MHz) δ: 7.84–7.81 (m, 2H), 7.58–7.56 (m, 3H), 0.25 (s, 9H) ppm. ^13^C NMR (CDCl_3_, 100 MHz) δ = 143.7, 131.9, 131.8, 129.7, 129.4, 129.1, 128.6, 125.3, 111.3, 100.1, −0.73 ppm. HRMS calcd. for C_21_H_23_N_3_O_5_S_2_ + H^+^ (M + H)^+^ 462.1157, found 462.1162.

#### *p*-Methylphenyl 3-deoxy-3-[4-phenylsulfane-1*H*-1,2,3-triazol-1-yl] 1-thio-β-D-galactopyranoside **4a**

To a suspension of **2** (20 mg, 0.046 mmol), CuI (1 mg, 10 mol%), and **3a** (9 mg, 0.069 mmol) in DCM (3 mL), was added DIPEA (15 μL, 0.069 mmol). The reaction mixture was stirred at room temperature for 2 days, then filtered, the filtrate was washed with methanol (10 mL), the solvent was concentrated, and the residue was purified with flash chromatography (SiO_2_, 5% MeOH in DCM) followed by preparative HPLC to afford **4a** (16 mg, 61%) as a white amorphous solid. [α]_D_^25^ + 43.7 (c 0.8, CH_3_OH). ^1^H NMR (CD_3_OD, 400 MHz): 8.24 (s, 1H), 7.48 (d, 1H, *J* 8 Hz), 7.28–7.23 (m, 4H), 7.17 (m, 1H), 7.14 (d, 2H, *J* 8 Hz), 4.75 (d, 1H, *J* 9.2 Hz), 4.19 (t, 1H, *J* 10.4 Hz), 4.11 (d, 1H, *J* 2.8 Hz), 3.80–3.70 (m, 3H), 2.32 (s, 3H). ^13^C NMR (CD_3_OD, 100 MHz) δ = 138.8, 138.0, 137.1, 133.2, 131.6, 130.7, 130.2, 130.16, 129.4, 127.7, 91.7, 80.8, 69.5, 67.9, 62.3, 21.1 ppm. HRMS calcd. for C_21_H_23_N_3_O_4_S_2_ + H^+^ (M + H)^+^ 446.1208, found 446.1207. The analytical purity was 98.6%.

#### *p*-Methylphenyl 3-deoxy-3- [4-phenylsulfinyl-1*H*-1,2,3-triazole-1yl] 1-thio-β-D-galactopyranoside **4b**

To a suspension of **2** (20 mg, 0.046 mmol), CuI (1 mg, 10 mol%), and **3b** (10 mg, 0.069 mmol) in DCM (3 mL), DIPEA (15 μL, 0.069 mmol) was added. The mixture was stirred at room temperature for 16 h, filtered, and washed with methanol (10 mL). The solvent was concentrated and the residue was purified with flash chromatography (SiO_2_, 5% MeOH in DCM), followed by preparative HPLC to afford **4b** (17 mg, 61%) as white amorphous solid. [α]_D_^25^ + 22.9 (c 0.7, CH_3_OH). ^1^H NMR (CD_3_OD, 400 MHz) δ = 8.33 and 8.31 (2s, 1H), 7.79–7.77 (m, 2H), 7.60–7.59 (m, 3H), 7.48–7.46 (m, 2H), 7.15–7.13 (m, 2H), 4.88 (m, 1H), 4.72 (dd, 1H, *J* 2 Hz, J 8 Hz), 4.13 (m, 1H), 4.06 (dd, 1H, *J* 2 Hz, *J* 6 Hz), 3.78 – 3.65 (m, 3H), 2.32 (s, 3H) ppm. ^13^C NMR (CD_3_OD, 100 MHz) δ = 151.42, 151.40, 143.4, 143.3, 138.8, 133.3, 133.2, 133.03, 133.0, 131.44, 131.42, 130.70, 130.69, 130.64, 126.7, 126.6, 126.1, 126.0, 91.54, 91.48, 80.7, 69.6, 69.3, 69.2, 67.8, 62.2, 21.1 ppm. HRMS calcd. for C_21_H_23_N_3_O_5_S_2_ + H^+^ (M + H)^+^ 462.1157, found 462.1162. The analytical purity was 96.1%.

#### *p*-Methylphenyl 3-deoxy-3- [4-phenylsulfonyl-1*H*-1,2,3-triazole-1yl] 1-thio-β-D-galactopyranoside **4c**

To a suspension of **2** (15 mg, 0.034 mmol), CuI (1 mg, 10 mol%), and **3c** (9 mg, 0.052 mmol) in DCM (3 mL), DIPEA (10 μL, 0.052 mmol) was added. The reaction mixture was stirred at room temperature for 2 h and then filtered and washed with methanol (10 mL). The solvent was concentrated and the residue was purified with flash chromatography (SiO_2_, 5% MeOH in DCM), followed by preparative HPLC to afford **4c** (16 mg, 72%) as white amorphous solid. [α]_D_^25^ + 56.8 (c 0.8, CH_3_OH). ^1^H NMR (CD_3_OD, 400 MHz) δ = 8.69 (s, 1H), 8.06–8.04 (m, 2H), 7.68 (m, 1H), 7.61 (m, 1H), 7.49–7.47 (m, 2H), 7.15 (d, 2H, *J* 8.0 Hz), 4.91 (dd, 1H, *J* 2.8 Hz, *J* 10.4 Hz), 4.73 (d, 1H, *J* 9.6 Hz), 4.18 (t, 1H, *J* 10.4 Hz), 4.06 (d, 1H, *J* 3.2 Hz), 3.76–3.66 (m, 3H), 2.33 (s, 3H) ppm. ^13^C NMR (CD_3_OD, 100 MHz) δ = 149.3, 142.0, 138.9, 135.2, 133.3, 131.5, 130.7, 130.6, 128.9, 128.2, 91.6, 80.7, 69.2, 67.8, 62.2, 21.1 ppm. HRMS calcd. for C_21_H_23_N_3_O_6_S_2_ + H^+^ (M + H)^+^ 478.1107, found 478.1105. The analytical purity was 94.2%.

#### 3′-Deoxy-3′- [4-phenylsulfane-1*H*-1,2,3-triazol-1-yl] -β-D-galactopyranosyl-1-thio-β-d-glucopyranoside **6a**

To a suspension of **5** (50 mg, 0.13 mmol), **3a** (26 mg, 0.19 mmol), and CuI (1 mg, 10 mol%) in acetonitrile (3 mL), DIPEA (35 μL, 0.19 mmol) was added. The reaction was stirred at room temperature overnight, filtered, and washed with methanol (10 mL); the solvent was evaporated, and the residue was purified by preparative HPLC to afford **6a**. (24 mg, 37%). [α]_D_^25^ + 37.1 (c 1.0, CH_3_OH). ^1^H NMR (CD_3_OD, 400 MHz) δ = 8.29 (s, 1H), 7.30 – 7.24 (m, 4H), 7.20 (m, 1H), 4.94 (dd, 1H, *J* = 2.8 Hz, *J* = 10.4 Hz), 4.77 (d, 1H, *J* 9.6 Hz), 4.25 (t, 1H *J* = 9.6 Hz), 4.10 (d, 1H *J* = 2.8 Hz), 3.90 (dd, 1H, *J* = 2.0 Hz, *J* = 12.0 Hz), 3.83–3.76 (m, 2H), 3.67–3.63 (m, 2H), 3.43–3.28 (m, 5H) ppm. ^13^C NMR (CD_3_OD, 100 MHz) δ = 138.0, 137.1, 130.2, 129.4, 127.7, 86.0, 84.5, 82.2, 81.2, 79.6, 74.7, 71.5, 69.7, 69.4, 69.39, 63.0, 62.6 ppm. HRMS calcd. for C_20_H_27_N_3_O_9_S_2_ + H^+^ (M + H)^+^ 518.1267, found 518.1274. The analytical purity was 87%.

#### 3′-Deoxy-3′- [4-phenylsulfinyl-1*H*-1,2,3-triazol-1-yl] -β-D-galactopyranosyl-1-thio-β-d-glucopyranoside **6b**

To a suspension of **5** (50 mg, 0.13 mmol), **3b** (27 mg, 0.19 mmol), and CuI (1 mg, 10 mol%) in 3 mL of acetonitrile, DIPEA (35 μL, 0.19 mmol) was added. The mixture was stirred at room temperature for overnight. The reaction mixture was filtered, the solids were washed with methanol (10 mL), the solvent was evaporated, and the residue purified by preparative HPLC to afford **6b**. (28 mg, 40%). [α]_D_^25^ + 36.5 (c 0.7, CH_3_OH). ^1^H NMR (400 MHz, CD_3_OD) δ = 8.38 and 8.36 (2s, 1H), 7.83–7.79 (m, 2H), 7.64–7.60 (m, 3H), 4.76 (m, 2H), 4.21 (q, *J* 10 Hz, 1H), 4.06 (dd, *J* 3.2 Hz, *J* 6.0 Hz, 1H), 3.89 (dt, *J* 1.6 Hz, *J* 12.0 Hz, 1H), 3.82 – 3.74 (m, 2H), 3.70–3.62 (m, 2H), 3.42–3.37 (m, 1H) ppm. ^13^C NMR (100 MHz, CD_3_OD) δ = 130.13, 130.10, 127.80, 127.79, 123.8, 123.2, 123.1, 83.0, 81.6, 79.3, 78.2, 76.7, 71.7, 68.6, 66.6, 65.5, 60.1, 59.6 ppm. HRMS calcd. for C_20_H_27_N_3_O_10_S_2_ + H^+^ (M + H)^+^ 534.1216, found 534.1221. The analytical purity was 93.3%.

#### 3′-Deoxy-3′- [4-phenylsulfonyl-1*H*-1,2,3-triazol-1-yl] -β-D-galactopyranosyl-1-thio-β-d-glucopyranoside **6c**

To a suspension of **5** (50 mg, 0.13 mmol), **3b** (27 mg, 0.19 mmol), and CuI (1 mg, 10 mol%) in acetonitrile (3 mL), DIPEA (35 μL, 0.19 mmol) was added. The mixture was stirred at room temperature overnight and filtered, the solids were washed with methanol (10 mL), the solvent was evaporated, and the residue was purified by preparative HPLC to afford **6b**. (28 mg, 40%). [α]_D_^25^ + 45.2 (c 1.1, CH_3_OH). ^1^H NMR (CD_3_OD, 400 MHz) δ = 8.75 (s, 1H), 8.09–8.06 (m, 2H), 7.71 (tt, 1H, *J* 1.2 Hz, *J* 8 Hz), 7.63 (tt, 2H, *J* 1.2 Hz, *J* 8 Hz), 4.94 (dd, 1H, *J* 2.8 Hz, *J* 10.4 Hz), 4.77 (d, 1H, *J* 9.6 Hz), 4.27 (t, 1H *J* 9.6 Hz), 4.07 (d, 1H *J* 2.8 Hz), 3.90 (dd, 1H, *J* 2 Hz, *J* 12 Hz), 3.82–3.76 (m, 2H), 3.67–3.63 (m, 2H), 3.43–3.28 (m, 5H). ^13^C NMR (CD_3_OD, 100 MHz) δ = 149.3, 142.1, 135.2, 130.6, 129.9, 128.2, 85.9, 84.5, 82.2, 81.1, 79.6, 74.7, 71.5, 69.6, 69.4, 68.3, 63.0, 62.5 ppm. HRMS calcd. for C_20_H_27_N_3_O_11_S_2_ + H^+^ (M + H)^+^ 550.1165, found 550.1163. The analytical purity was 92.2%.

### Fluorescence Polarization

Fluorescence polarization experiments were carried out either with a POLARStar plate reader and FLUOstar Galaxy software or with a PheraStarFS plate reader and PHERAstar Mars version 2.10 R3 software (BMG, Offenburg, Germany). The dissociation constant (*K*_d_) values were determined in PBS as described earlier.^13,18^ Compounds were dissolved in neat DMSO at 20 mM and diluted in PBS to 3–6 different concentrations to be tested in duplicate. Average *K*_d_ values and SEMs were calculated from 2 to 8 single-triple point measurements showing between 20 and 80% inhibition.

### Co-Crystallization of Galectin-3C with Compounds **6a**, **6b**, and **6c**

Small crystals of lactose-bound galectin-3C were grown with the hanging drop method in NeXtal plates (NeXtal Biotechnologies) with the following reservoir condition: 20% (w/v) PEG 4000, Tris-HCl pH 7.5, 0.4 M NaSCN, 10 mM β-mercaptoethanol, as described previously.^5^ Small crystals were then moved to drops containing the same reservoir with the addition of 10 mM of the ligand (**6a**, **6b**, and **6c**), from a 100 mM stock solution in DMSO. Soaking lasted for 12–15 h. 10% PEG 400 was added as a cryoprotectant just before freezing the crystals. Soaked crystals were then frozen in liquid nitrogen.

### Data Collection and Structure Solution of Galectin-3C in Complex with **6a**, **6b**, and **6c**

Data were collected at the BioMAX beamline of the MAX IV synchrotron in Lund, Sweden. For each complex, 3600 diffraction images were collected with a 0.1° rotation and 11 ms exposure time. All data were integrated using XDS. Scaling and merging were done with Aimless.^19^ Cross-validation of refinement was based on 10% of the reflections. Molecular replacement was done using Phaser in the Phenix suite^20^ version 1.14 using the high-resolution lactose – galectin-3C structure^5^ (PDB ID 3ZSJ), with lactose and water molecules removed, as the template. Ligands and their crystallographic restraints were generated through phenix.eLBOW.^21^ Restrained refinement was then performed using phenix.refine. Manual rebuilding, including the addition of water molecules, was done using Coot.^22^ All of the images were made with PyMOL (Schrödinger LLC).

### Isothermal Titration Calorimetry

All ITC experiments were performed on a MicroCal PEAQ-ITC (Malvern) at 301 K with 11 injections of 2.5 μL per injection (first injection, 0.4 μL) of the ligand into the protein. Stock solutions of the ligands were prepared in a buffer at 5 mM. The ligands were diluted with buffer to concentrations between 2 and 3 mM. All experiments were performed with galectin-3C concentrations between 0.19 to 0.25 mM in the cell. All thermograms were integrated using NITPIC, the titration curves were fitted using SEDPHAT with error estimates using the automatic confidence interval search with projection method, and all figures were made in GUSSI.

## Supporting Information Available

The crystal structures of galectin-3C in complex with compounds **6a, 6b,** and **6c** have been deposited in the Protein Data Bank (http://wwpdb.org) with accession numbers 8PBF, 8PF9, and 8PFF. The structures will be released on article publication (PDF)
Molecular formula strings (SMILE) as a csv file (CSV)

## Accession Codes

Protein Data Bank: 8PBF, 8PF9, and 8PFF.

## Abbreviations used

ADME: absorption, distribution, metabolism and excretion
CRD: carbohydrate recognition domain
DCM: dichloromethane
DIPEA: N,N-diisopropylethylamine
HPLC: high-pressure liquid chromatography
ITC: isothermal titration calorimetry
MD: molecular dynamics
mCPBA: meta-chloroperoxybenzoic acid
MS: mass spectrometry
NMR: nuclear magnetic resonance
RMSD: root-mean-square deviation
TLC: thin layer chromatography
UPLC: ultraperformance liquid chromatography
UV: ultraviolet
