# Supplementary material for: Ligand Sulfur Oxidation State Progressively Alters Galectin-3-Ligand Complex Conformations To Induce Affinity-Influencing Hydrogen Bonds
Source: J Med Chem. 2023 Oct 25;66(21):14716–23. doi: 10.1021/acs.jmedchem.3c01223 (PMC10641817; doi:10.1021/acs.jmedchem.3c01223)
Supplement: Supplementary file 1 — jm3c01223_si_001.pdf [file jm3c01223_si_001.pdf]

Supporting information

Ligand sulfur oxidation state progressively alters galectin-3-ligand complex conformations to induce affinity-influencing hydrogen bonds

Mukul Mahanti[Ψ,a] Kumar Bhaskar Pal[Ψ,a], Rohit Kumar[Ψ,b], Markus Schulze[a], Hakon Leffler[d], Derek T.Logan,[b,*] and Ulf J Nilsson[a*]

[a]Centre for Analysis and Synthesis, Department of Chemistry, Lund University, Box 124, SE-221 00 Lund, Sweden

[b]Centre for Molecular Protein Science, Department of Chemistry, Lund University, Box 124, SE-221 00 Lund, Sweden

[d]Department of Laboratory Medicine, Section MIG, Lund University BMC-C1228b, Klinikgatan 28, 221 84 Lund, Sweden

Ψ These authors contributed equally.

* to whom correspondence may be addressed: ulf.nilsson@chemistry.lu.se; derek.logan@biochemistry.lu.se

Table of Contents

.

1H NMR and 13C NMR spectra of compounds 3b, 4a-c, and 6a-c.................................... S3-S16

LC-MS summary and purities for compounds 4a-c and 6a S17

LC-MS data files for compounds 4a-c and 6a S18-S32

^{1}H NMR (CD_3OD, 400 Hz)

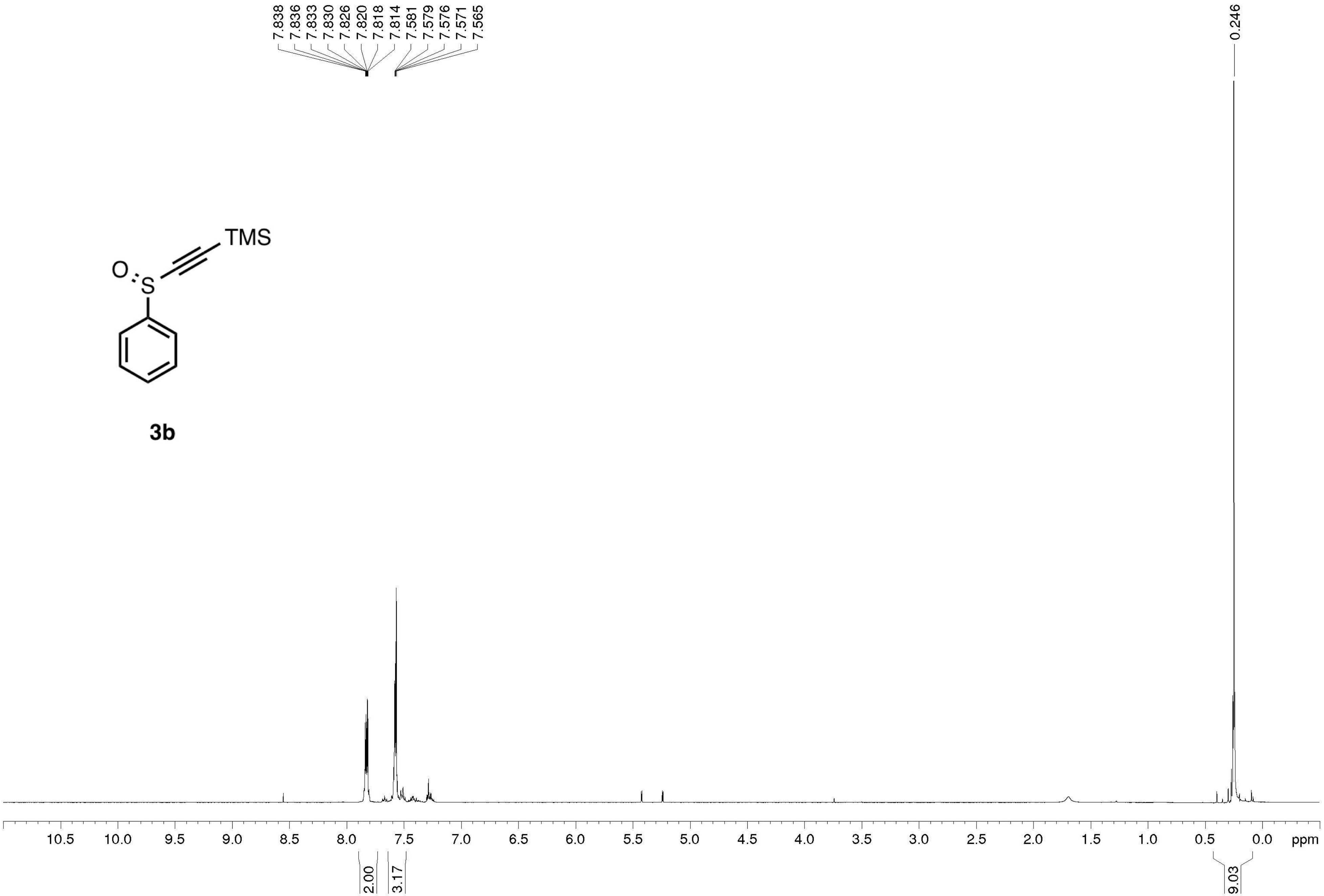

3b

^{13}C NMR (CD_3OD, 100 Hz)

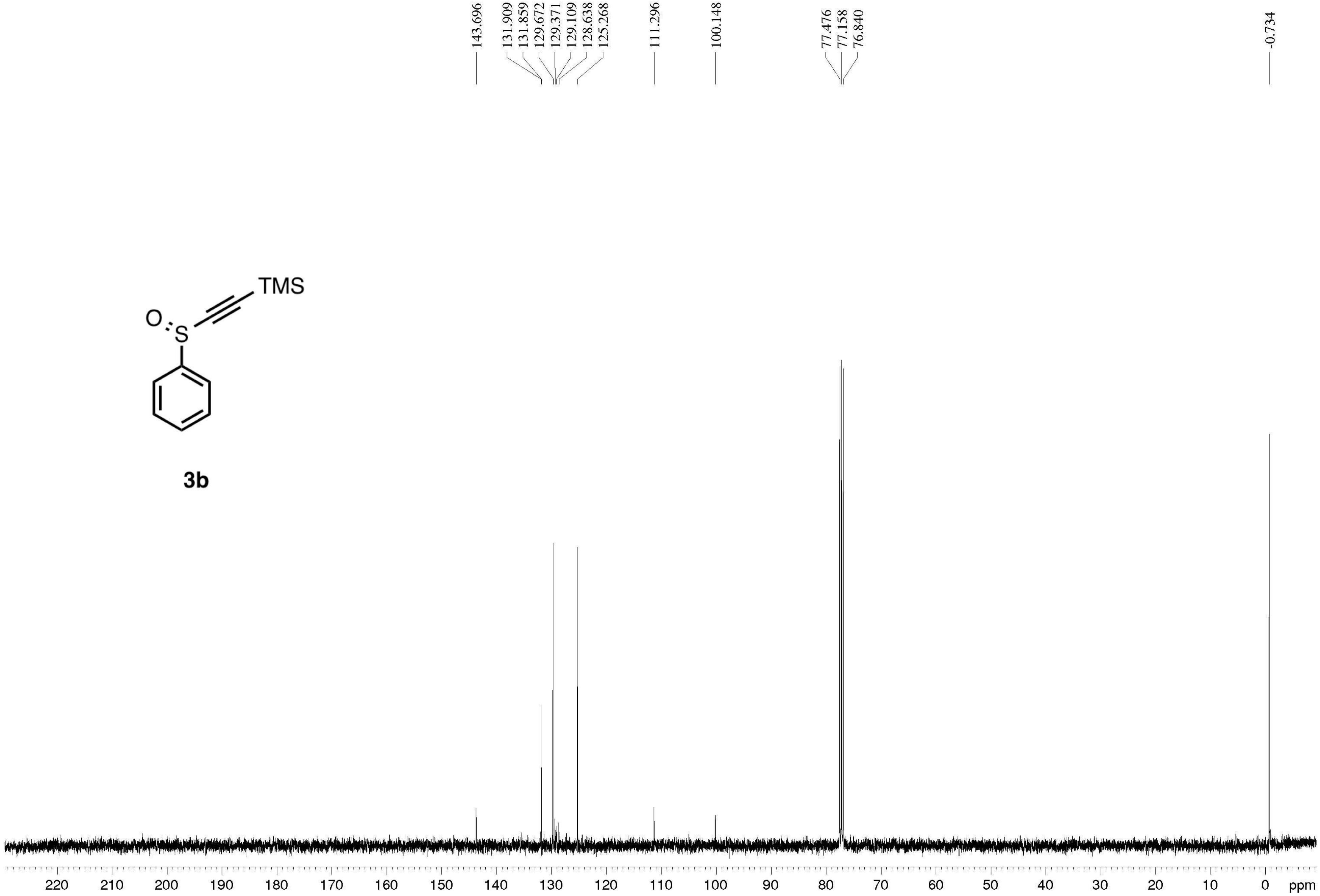

^{1}H NMR (CD_3OD, 400 MHz)

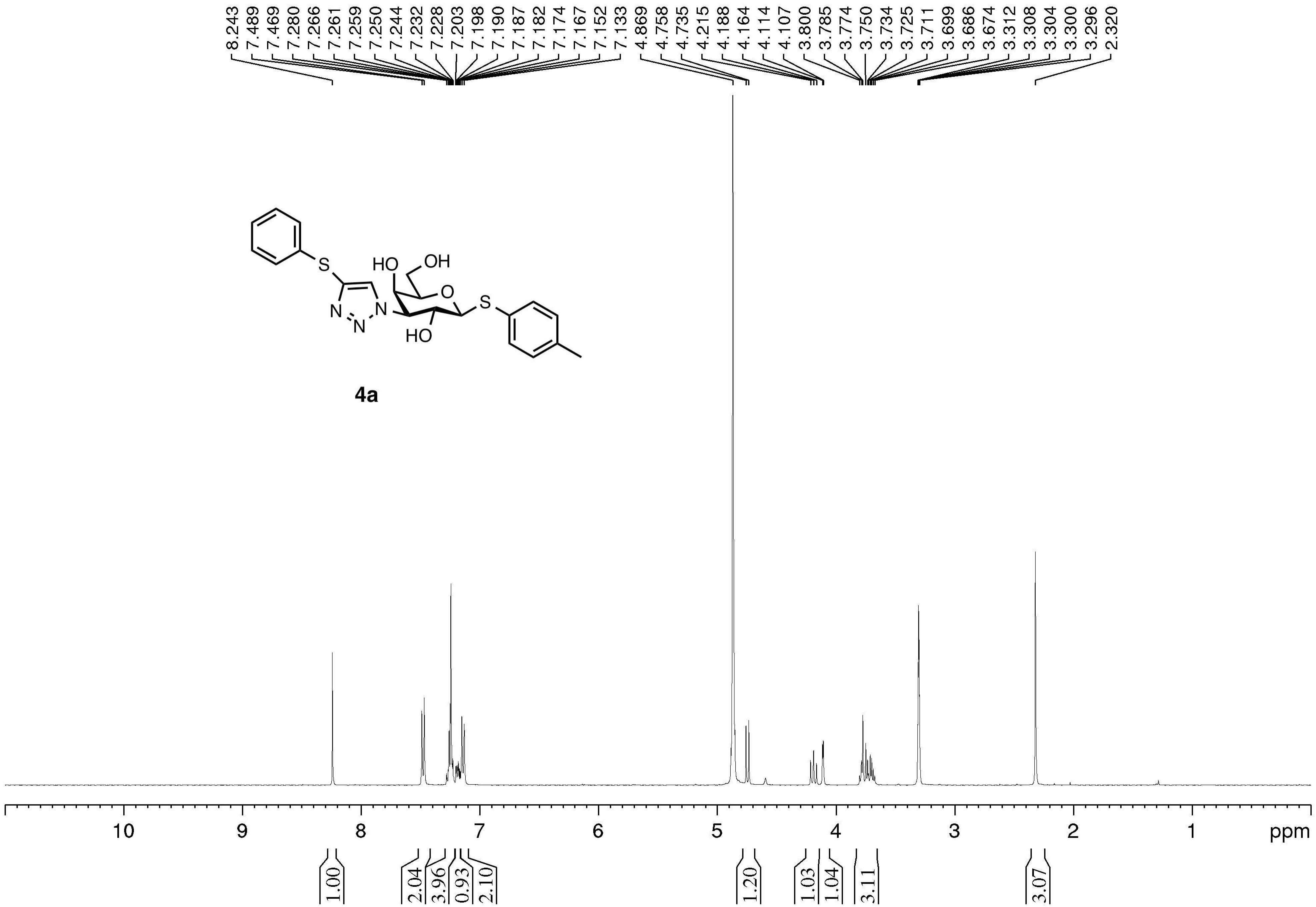

^{13}C NMR (CD_3OD, 100 MHz)

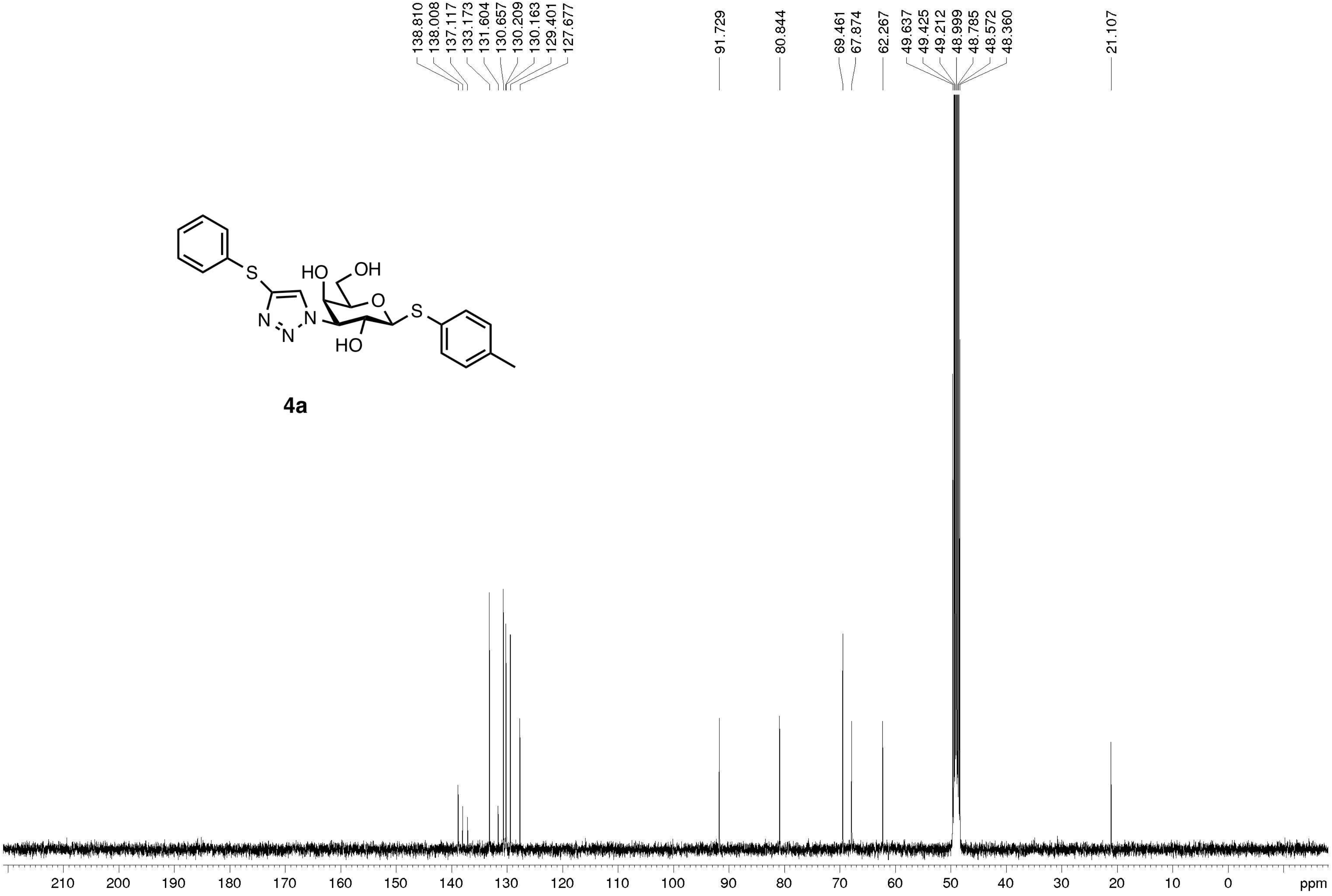

4a

^{1}H NMR (CD_3OD, 400 Hz)

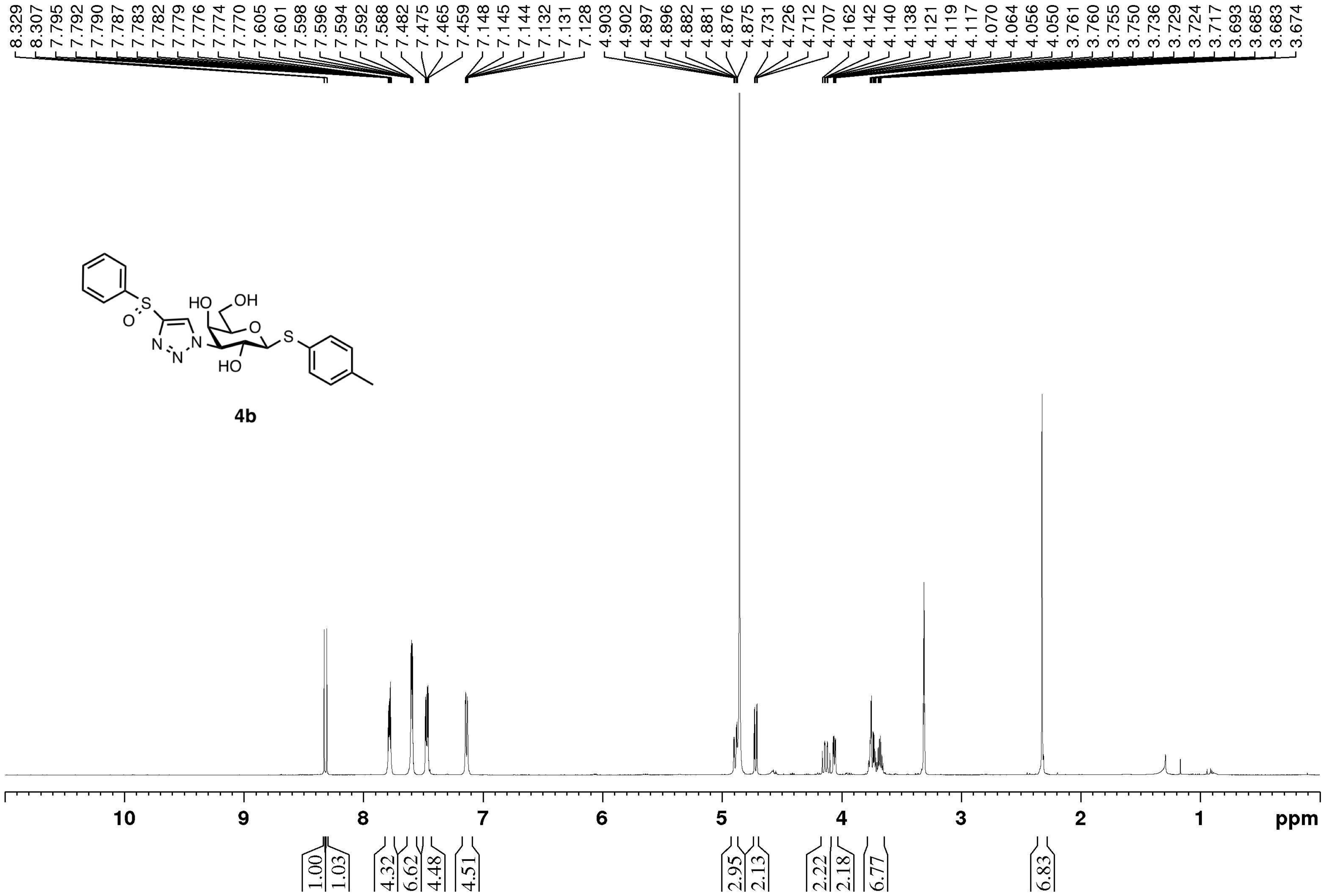

^{13}C NMR (CD_3OD, 100 Hz)

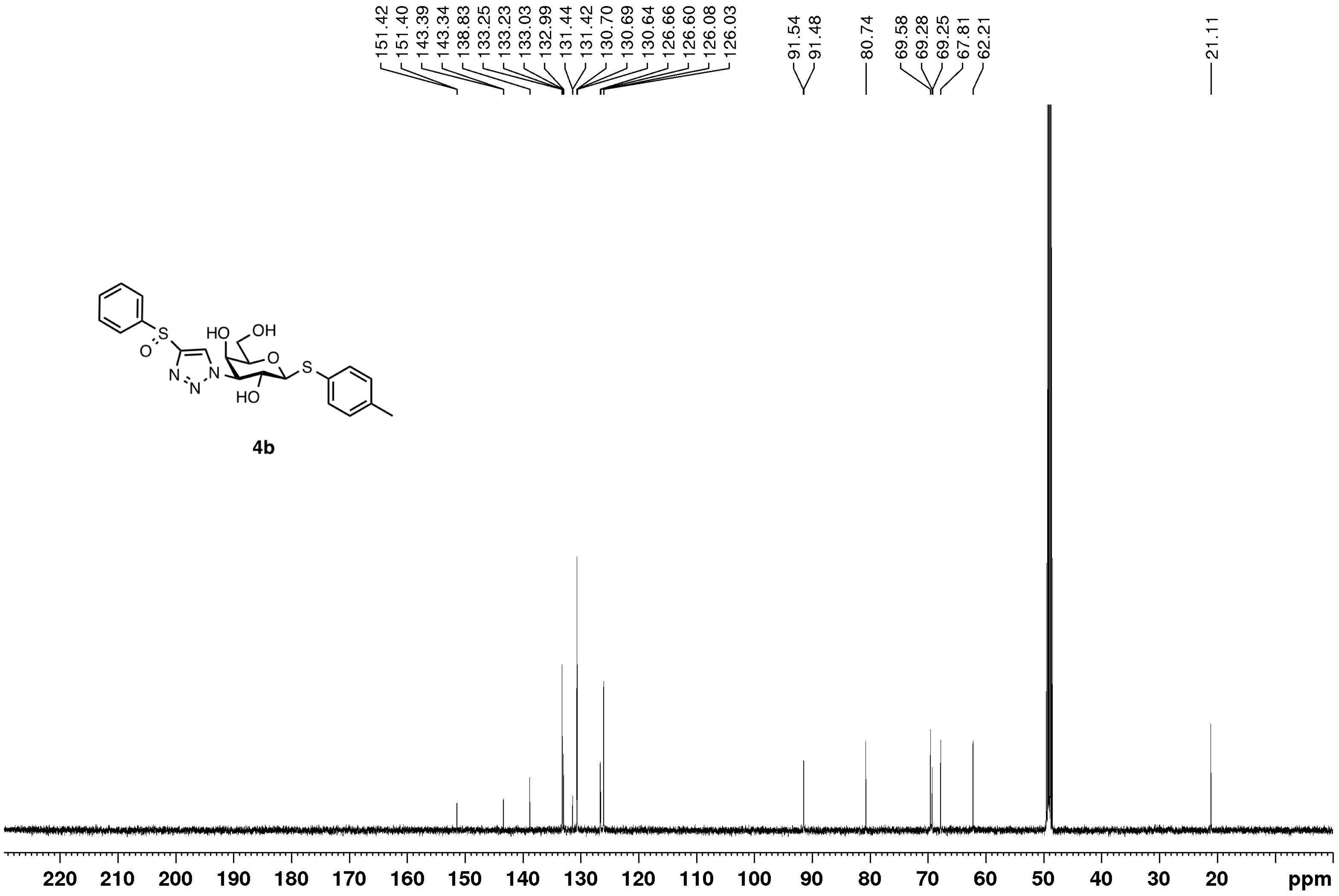

4b

^{1}H NMR (CD_3OD, 400 Hz)

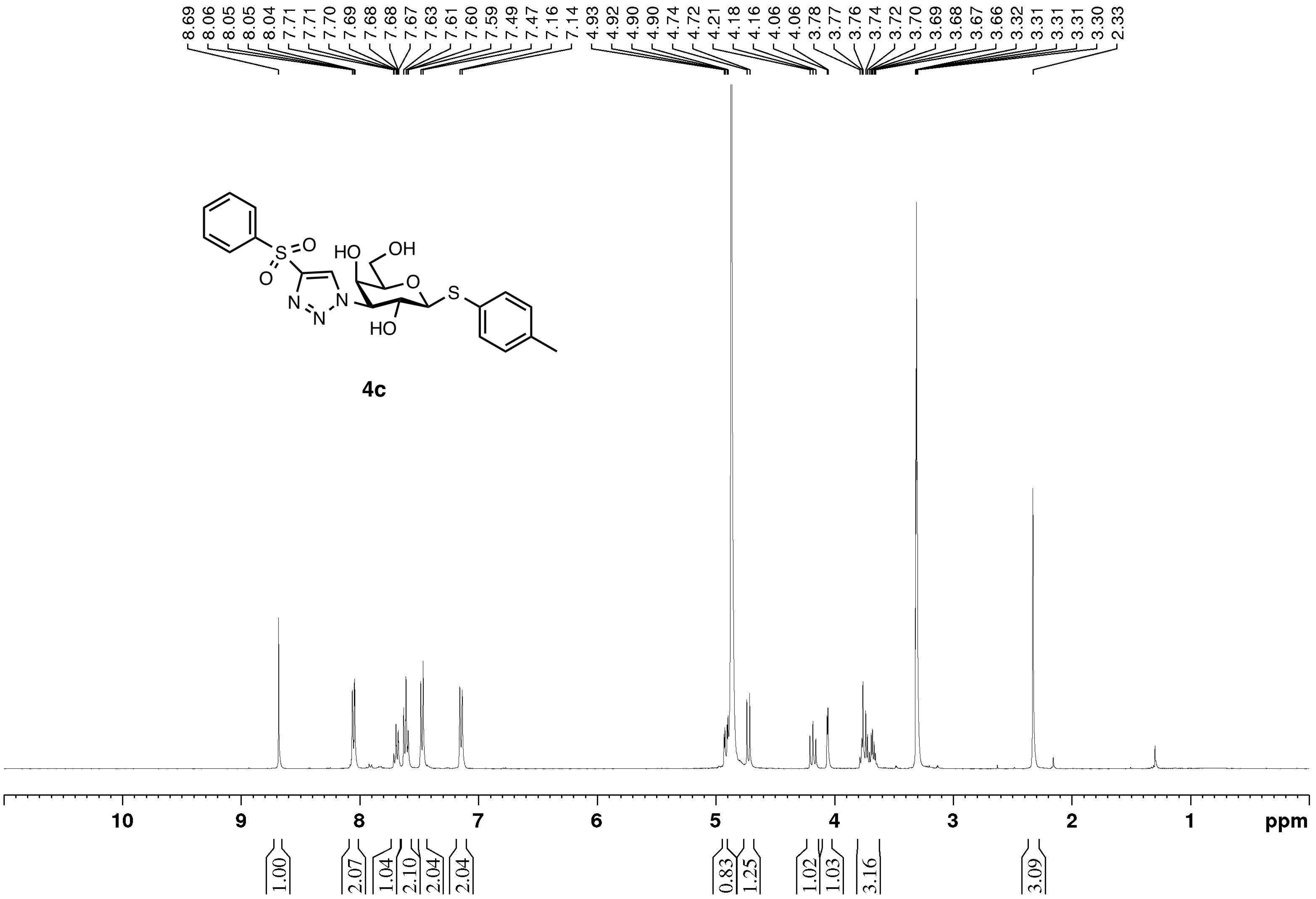

^{13}C NMR (CD_3OD, 100 Hz)

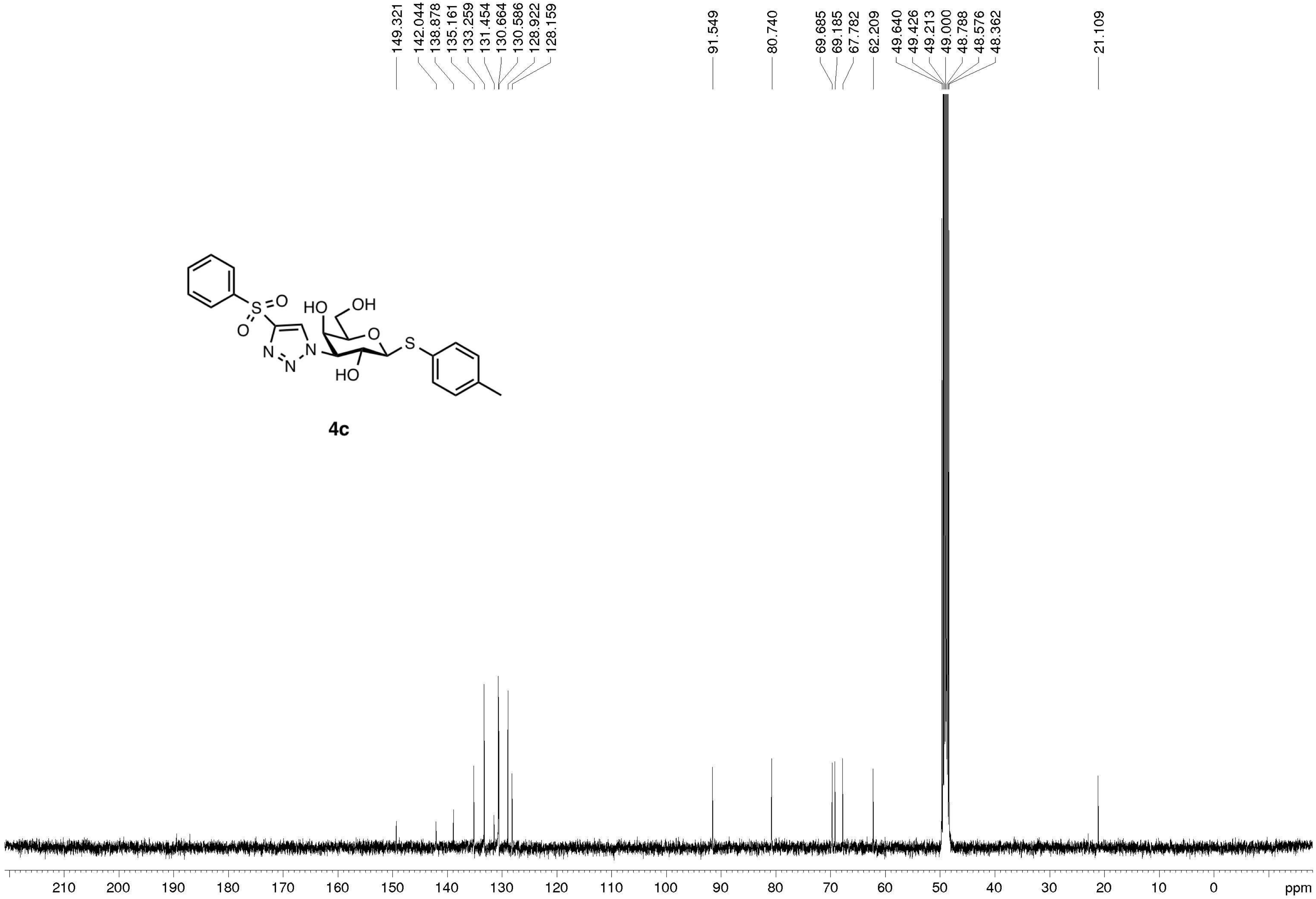

4c

^{1}H NMR (CD_3OD, 400 Hz)

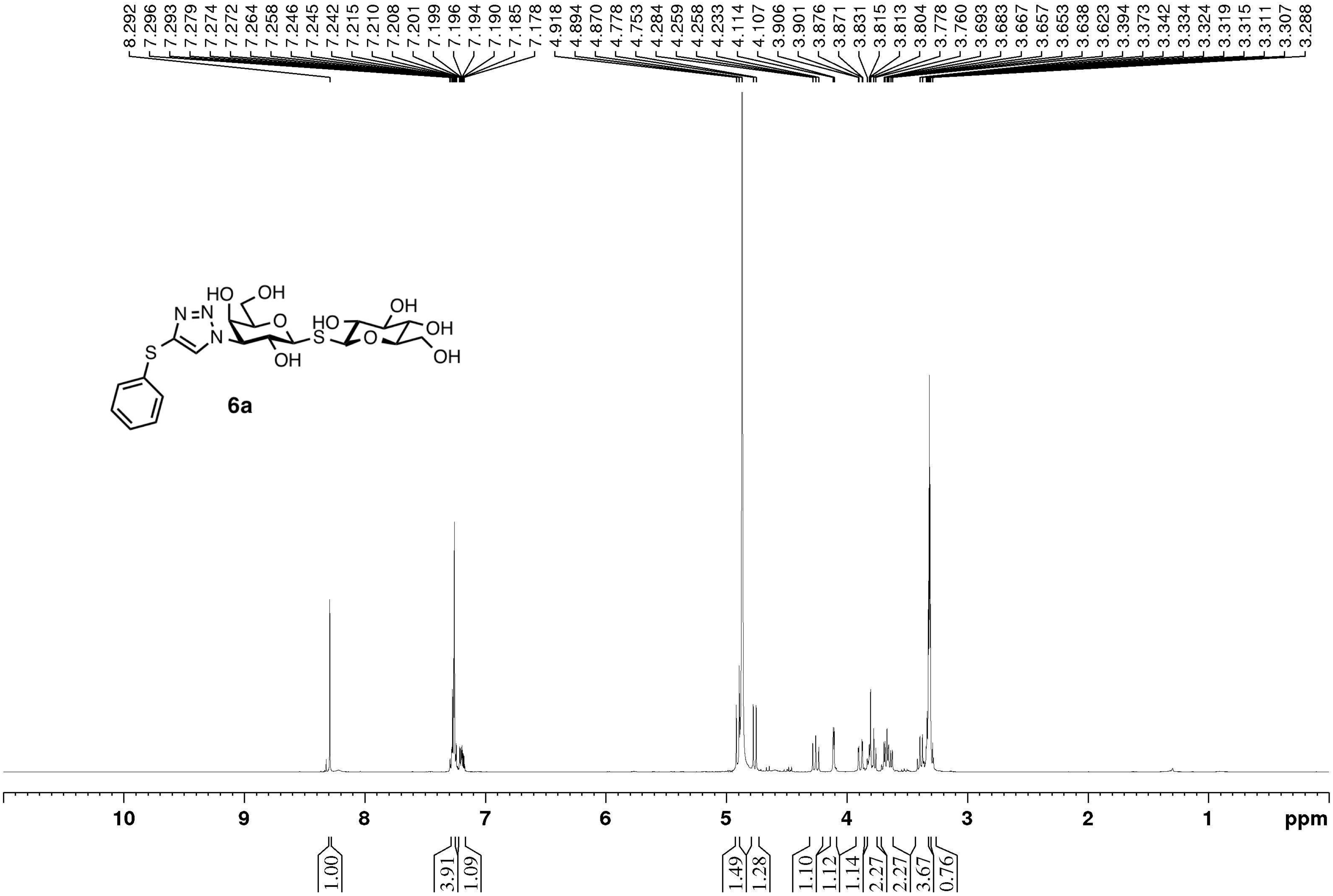

^{13}C NMR (CD_3OD, 100 Hz)

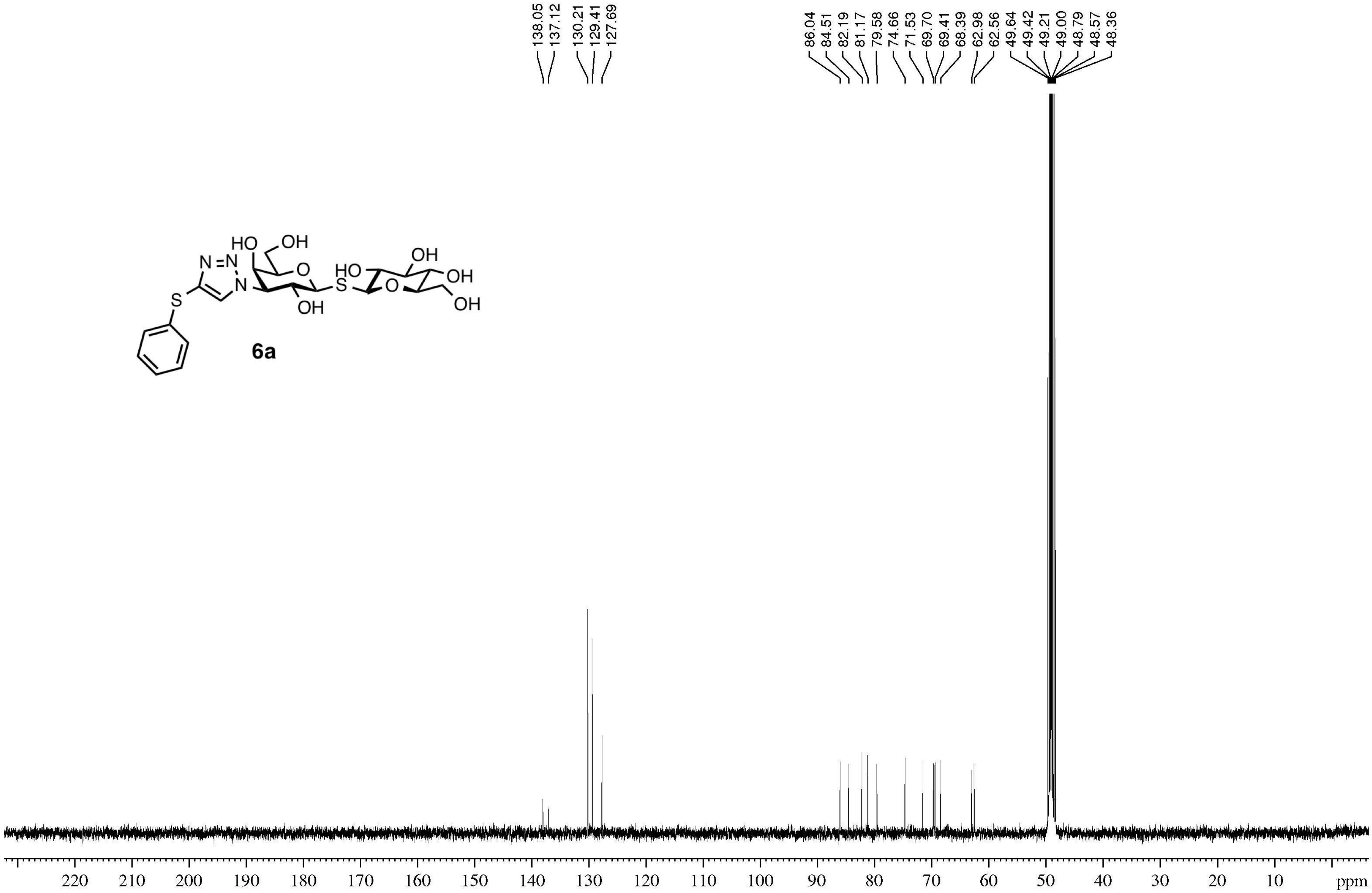

^{1}H NMR (CD_3OD, 400 Hz)

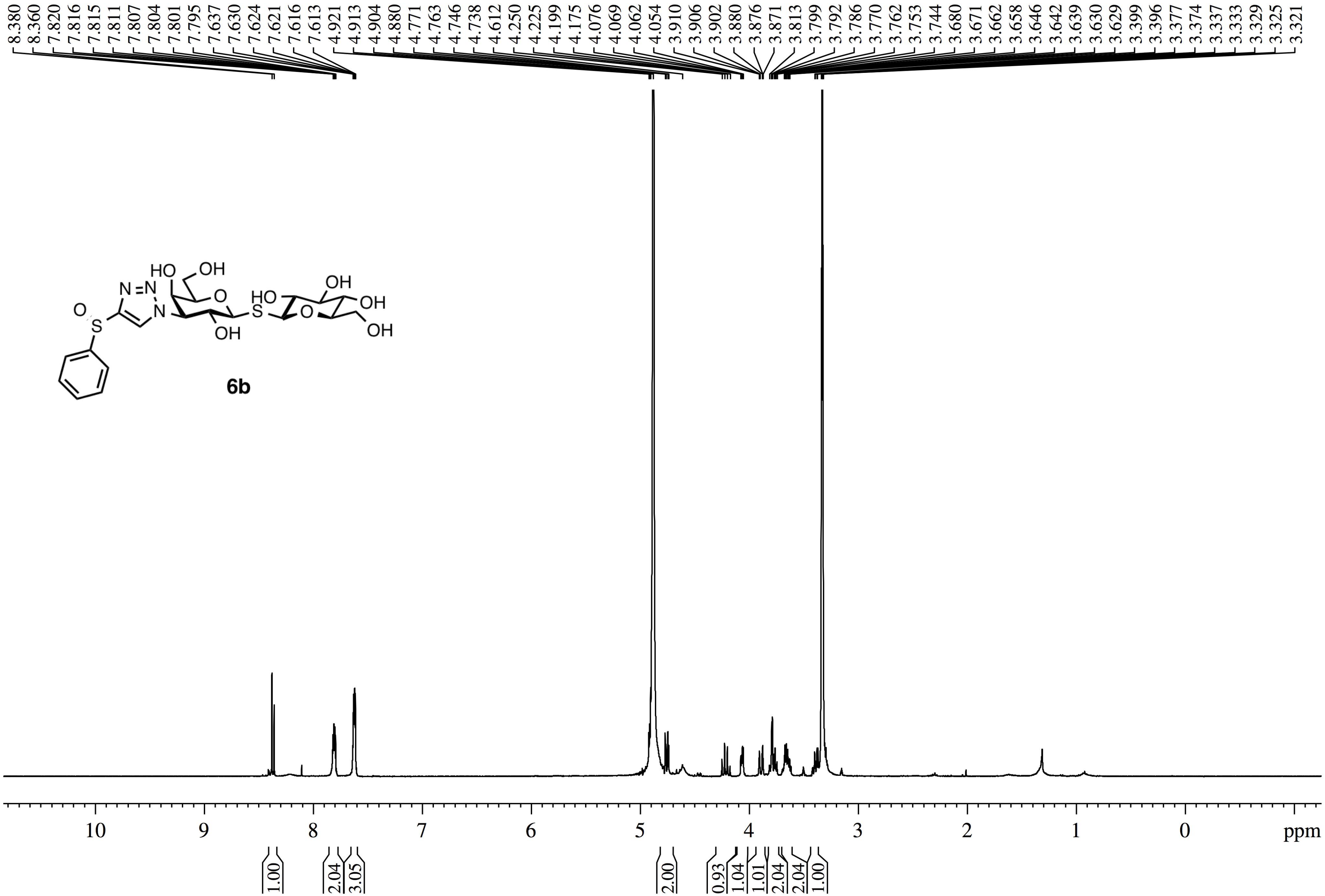

^{13}C NMR (CD_3OD, 100 Hz)

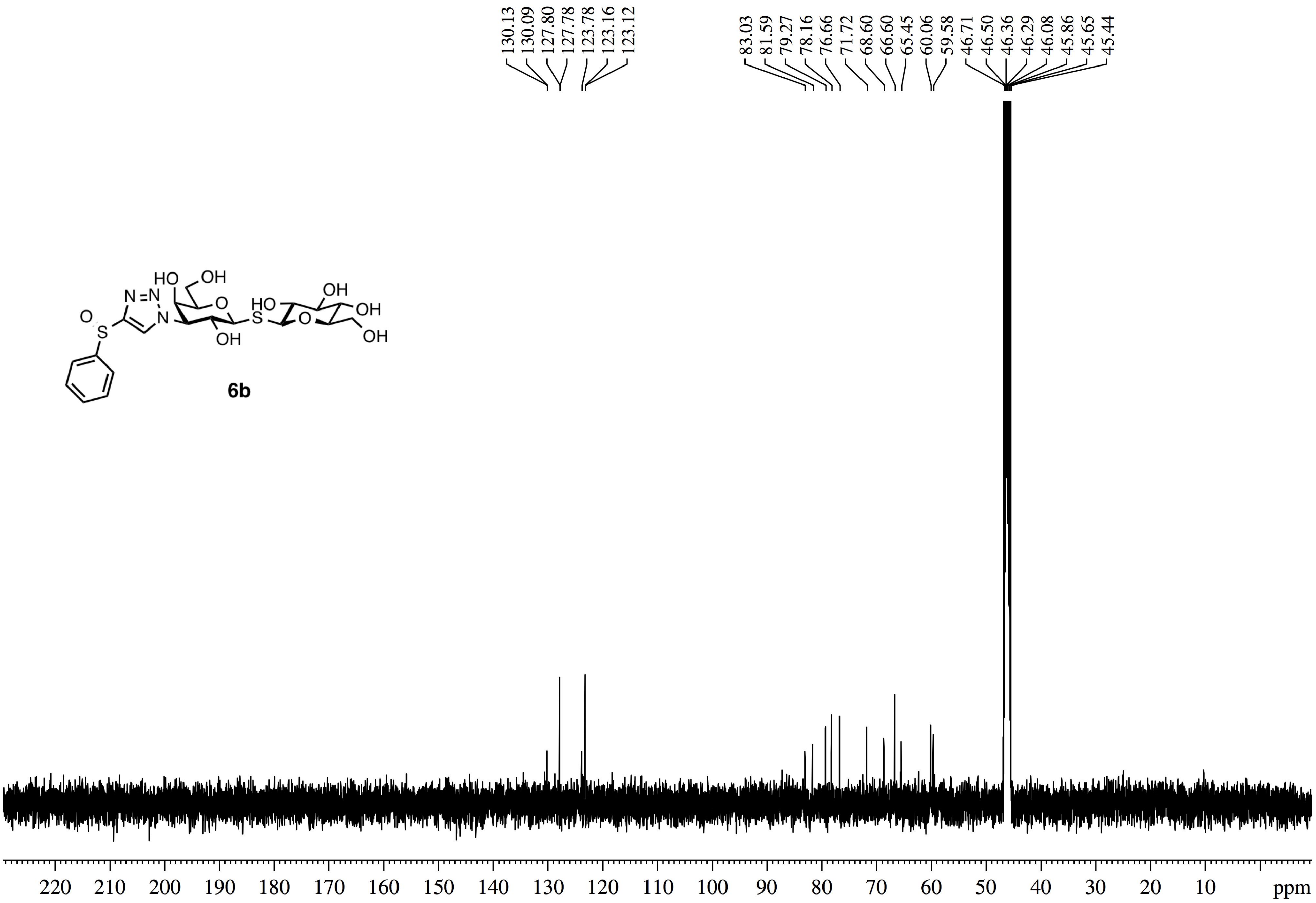

6b

^{1}H NMR (CD$_3$OD, 400 Hz)

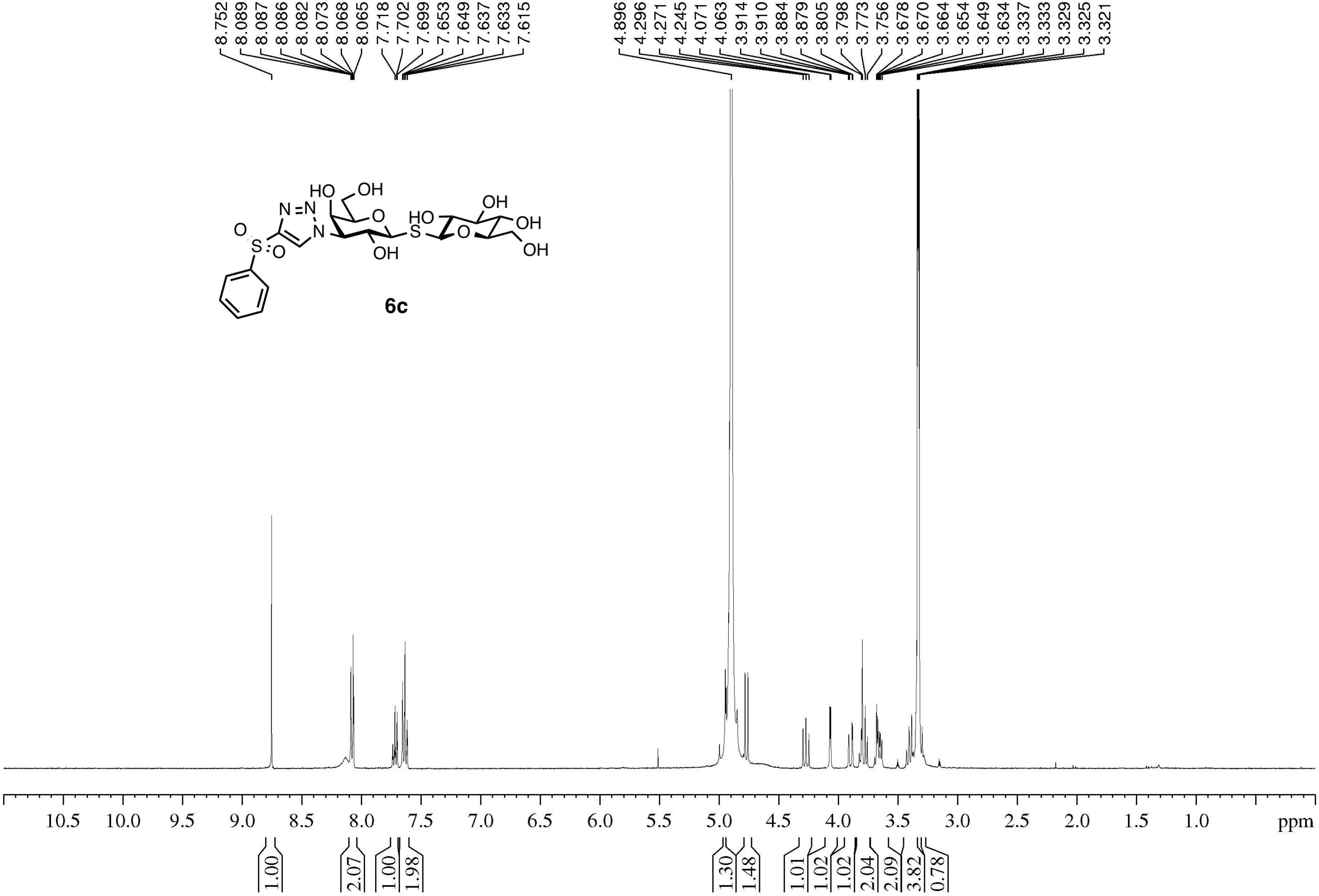

^{13}C NMR (CD_3OD, 100 Hz)

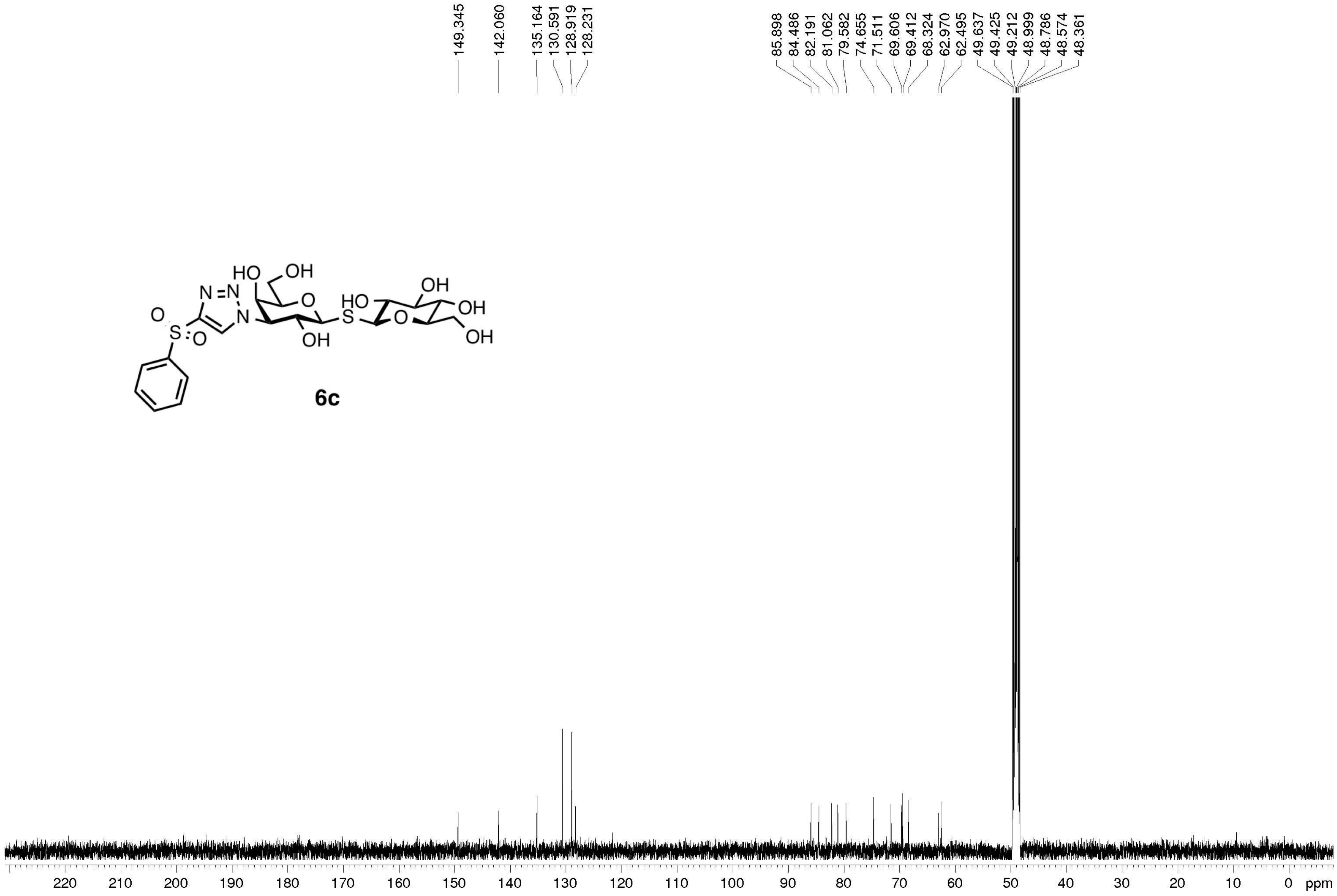

LC-HRMS Results

U**PLC MS:**
System: Waters Acquity UPLC + Waters XEVO-G2

MS methods:
ESI+: Cap 3.0 kV, Cone 40, **ESI-:** Cap 2.5 kV, Cone 30,
Both + & -: Ext 4, Source Temp 120, Des Temp 500, Cone gas 50, Des gas 800. , Centroid resolution mode, m/z 50-1200, Lockspray, LeuEnk m/z 556.2771 (+) or 554.2615, 0.25 s every 30 s, average 3,

Tune Page: LCMS_UN
MS Methods: UN_ESIpos_standard

LC Method Standard (Inlet Method:UN_A1_B1_10min_col1_PDA_STD)
Column: Waters Acquity CSH C18, 1.7 um, 2.1 x100 mm
MPA: 0.1% FA in Water and MPB: 0.1% FA in Acetonitrile, flow rate 0.50 mL/min, col temp 60°C
0-0.7min: 5% B, 0.7-10.0 min: 5-95% B, 10.0-11.0 min 95% B, 11.0-11.1 min 95-5%B, 11.1-13 min 3%B
1-3 uL injection, detection 190-300 nm

RESULT SUMMARY, see attachment for details: UV purity in area % at 254 nm.

Sample	Ion	Found m/z	Predicted m/z	UV purity (area %)	Comment
**4a**	$C_{21}H_{23}N_3O_{44}S_2 + H^+$	446.1207	446.1208	98.6%	
**4b**	$C_{21}H_{23}N_3O_5S_2 + H^+$	462.1162	462.1157	96.1%	
**4c**	$C_{21}H_{23}N_3O_6S_2 + H^+$	478.1105	478.1107	94.2%	
**6a**	$C_{20}H_{27}N_3O_9S_2 + H^+$	518.1274	518.1267	87%	
**6b**	$C_{20}H_{27}N_3O_{10}S_2 + H^+$	534.1221	534.1216	93.3%	
**6c**	$C_{20}H_{27}N_3O_{11}S_2 + H^+$	550.11163	550.1165	92.4%	

Sofia Essen, Lunds universitet, CAS

Compound: 4a

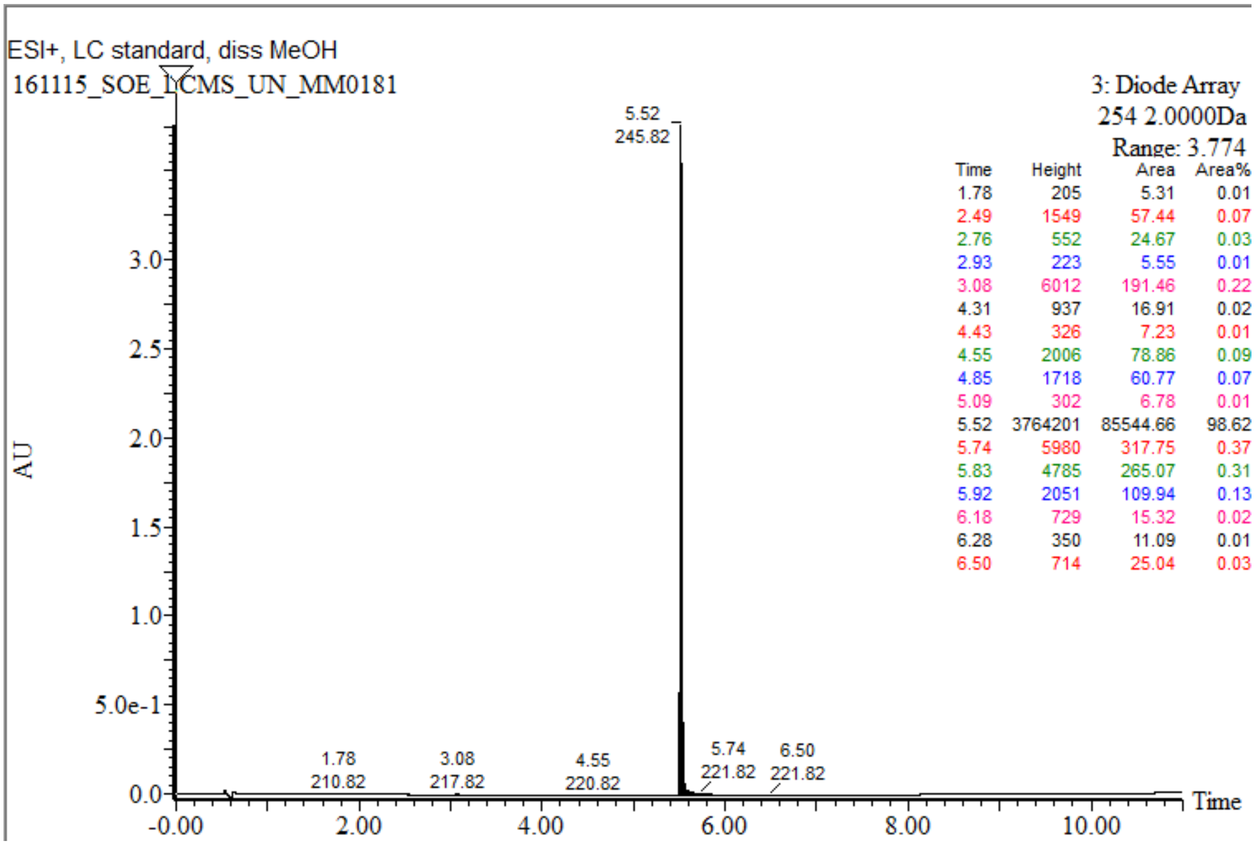

Time	Height	Area	Area%
1.78	205	5.31	0.01
2.49	1549	57.44	0.07
2.76	552	24.67	0.03
2.93	223	5.55	0.01
3.08	6012	191.46	0.22
4.31	937	16.91	0.02
4.43	326	7.23	0.01
4.55	2006	78.86	0.09
4.85	1718	60.77	0.07
5.09	302	6.78	0.01
5.52	3764201	85544.66	98.62
5.74	5980	317.75	0.37
5.83	4785	265.07	0.31
5.92	2051	109.94	0.13
6.18	729	15.32	0.02
6.28	350	11.09	0.01
6.50	714	25.04	0.03

Elements Used:
C: 0-100 H: 0-100 N: 0-8 O: 0-10 S: 1-2

Mass	Calc. Mass	mDa	PPM	DBE	Formula	Fit Conf %	C	H	N	O	S
446.1207	446.1208	-0.1	-0.2	11.5	C21 H24 N3 O4 S2	5.76	21	24	3	4	2
	446.1215	-0.8	-1.8	20.5	C29 H20 N O2 S	0.00	29	20	1	2	1
	446.1222	-1.5	-3.4	16.5	C22 H20 N7 S2	1.18	22	20	7		2
	446.1188	1.9	4.3	21.5	C25 H16 N7 S	0.02	25	16	7		1
	446.1233	-2.6	-5.8	7.5	C17 H24 N3 O9 S	93.04	17	24	3	9	1

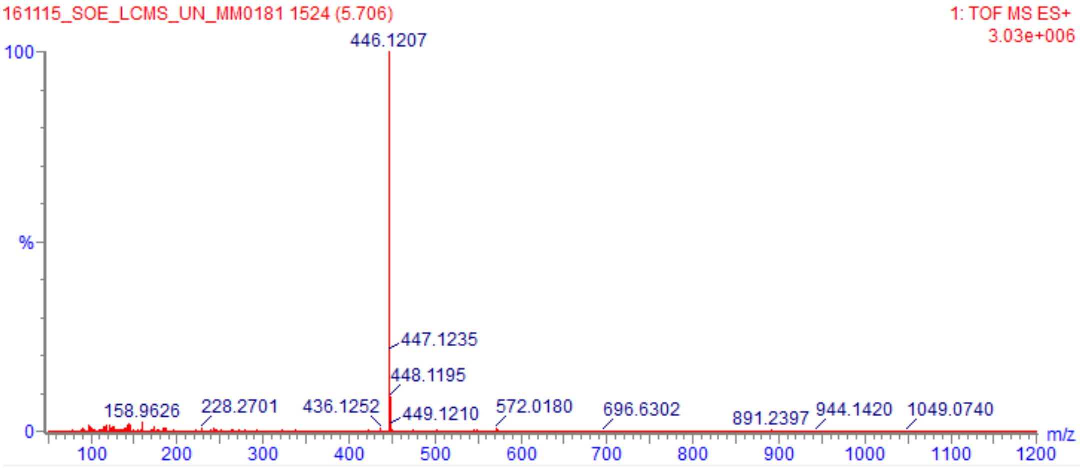

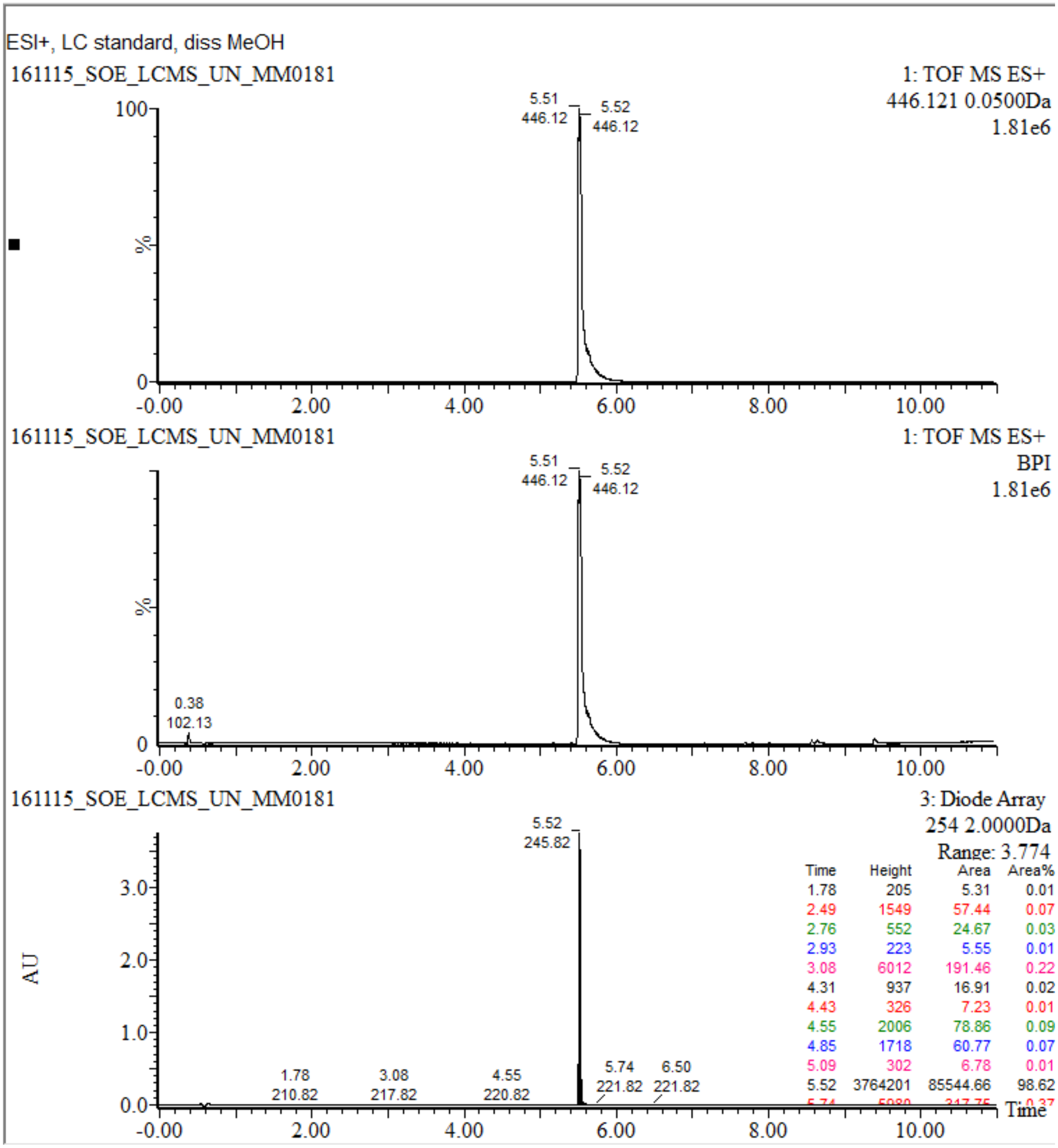
ESI+, LC standard, diss MeOH
161115_SOE_LCMS_UN_MM0181
1: TOF MS ES+
446.121 0.0500Da
1.81e6
100
%
0
5.51
446.12
5.52
446.12
-0.00
2.00
4.00
6.00
8.00
10.00
161115_SOE_LCMS_UN_MM0181
1: TOF MS ES+
BPI
1.81e6
5.51
446.12
5.52
446.12
%
0.38
102.13
0
161115_SOE_LCMS_UN_MM0181
3: Diode Array
254 2.0000Da
Range: 3.774
AU
3.0
2.0
1.0
0.0
5.52
245.82
1.78
210.82
3.08
217.82
4.55
220.82
5.74
221.82
6.50
221.82
Time Height Area Area%
1.78 205 5.31 0.01
2.49 1549 57.44 0.07
2.76 552 24.67 0.03
2.93 223 5.55 0.01
3.08 6012 191.46 0.22
4.31 937 16.91 0.02
4.43 326 7.23 0.01
4.55 2006 78.86 0.09
4.85 1718 60.77 0.07
5.09 302 6.78 0.01
5.52 3764201 85544.66 98.62
Time

Compound: 4b

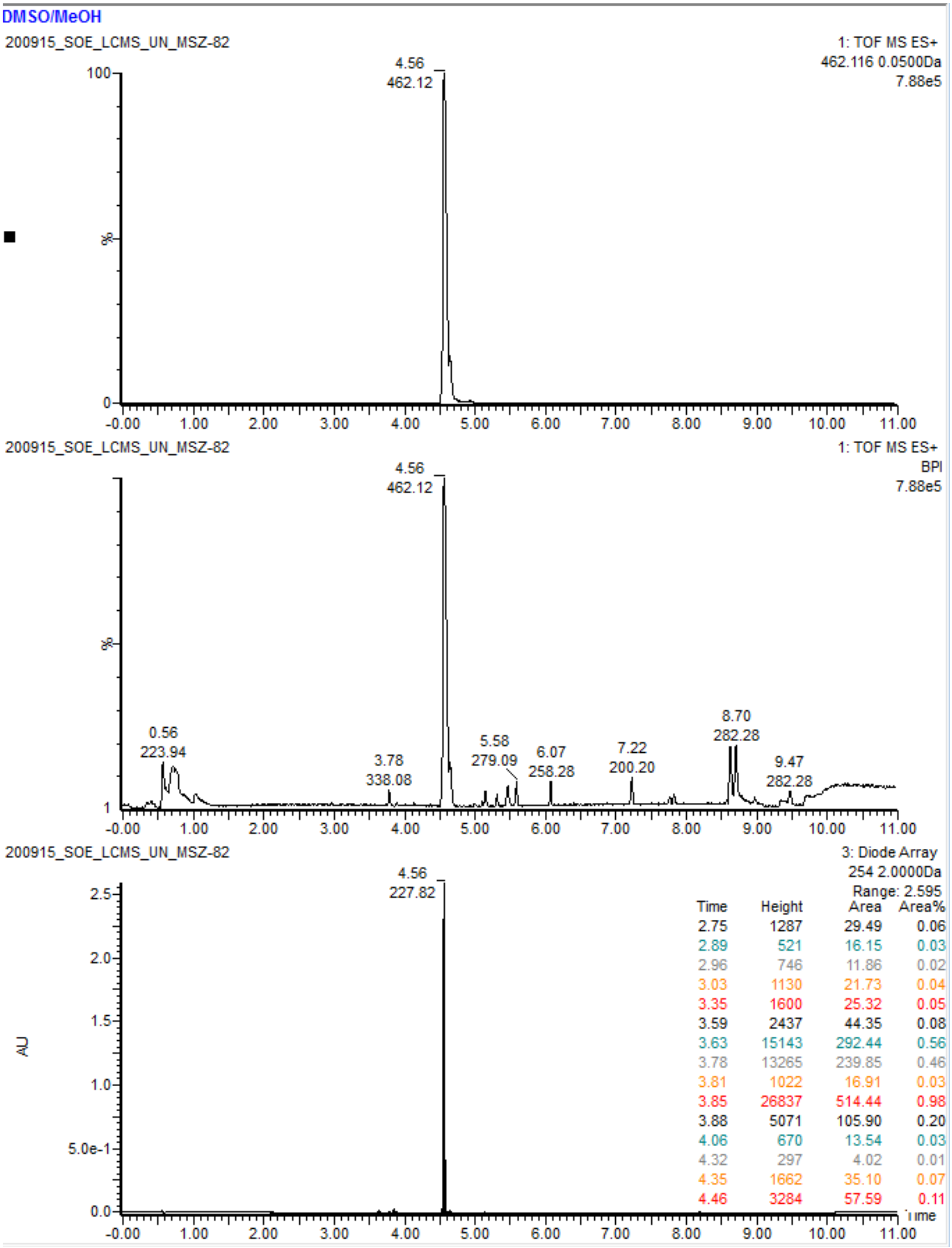

Time	Height	Area	Area%
2.75	1287	29.49	0.06
2.89	521	16.15	0.03
2.96	746	11.86	0.02
3.03	1130	21.73	0.04
3.35	1600	25.32	0.05
3.59	2437	44.35	0.08
3.63	15143	292.44	0.56
3.78	13265	239.85	0.46
3.81	1022	16.91	0.03
3.85	26837	514.44	0.98
3.88	5071	105.90	0.20
4.06	670	13.54	0.03
4.32	297	4.02	0.01
4.35	1662	35.10	0.07
4.46	3284	57.59	0.11

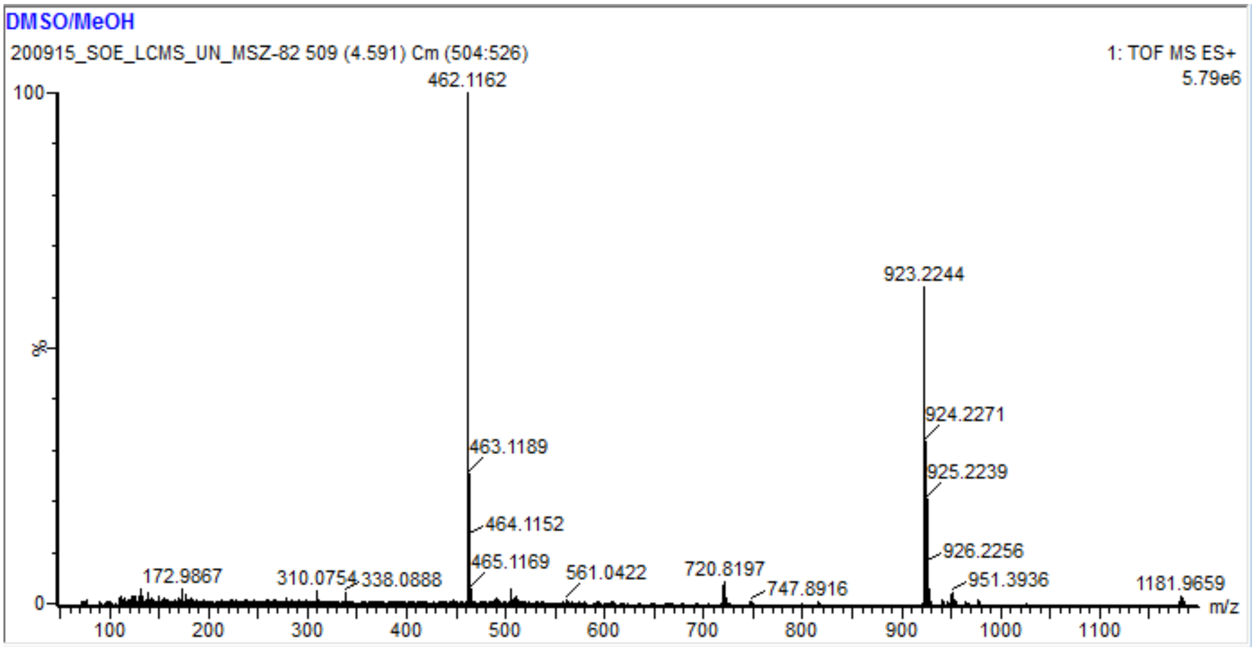

Single Mass Analysis

Tolerance = 2.0 mDa / DBE: min = -1.5, max = 50.0
Element prediction: Off
Number of isotope peaks used for i-FIT = 3
Monoisotopic Mass, Even Electron Ions
618 formula(e) evaluated with 2 results within limits (up to 10 closest results for each mass)

Elements Used:
C: 0-80 H: 0-100 N: 0-5 O: 2-8 S: 0-2

Mass	Calc. Mass	mDa	PPM	DBE	Formula	i-FIT	i-FIT Norm	Fit Conf %	C
462.1162	462.1164	-0.2	-0.4	20.5	C29 H20 N O3 S	38.5	17.745	0.00	29
	462.1157	0.5	1.1	11.5	C21 H24 N3 O5 S2	20.7	0.000	100.00	21

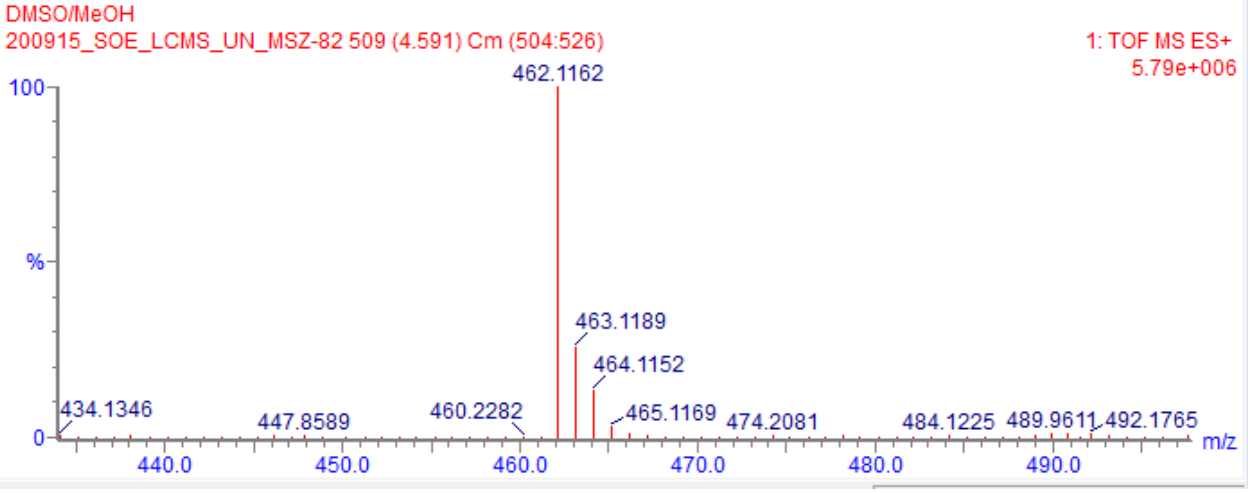

Compound: 4c

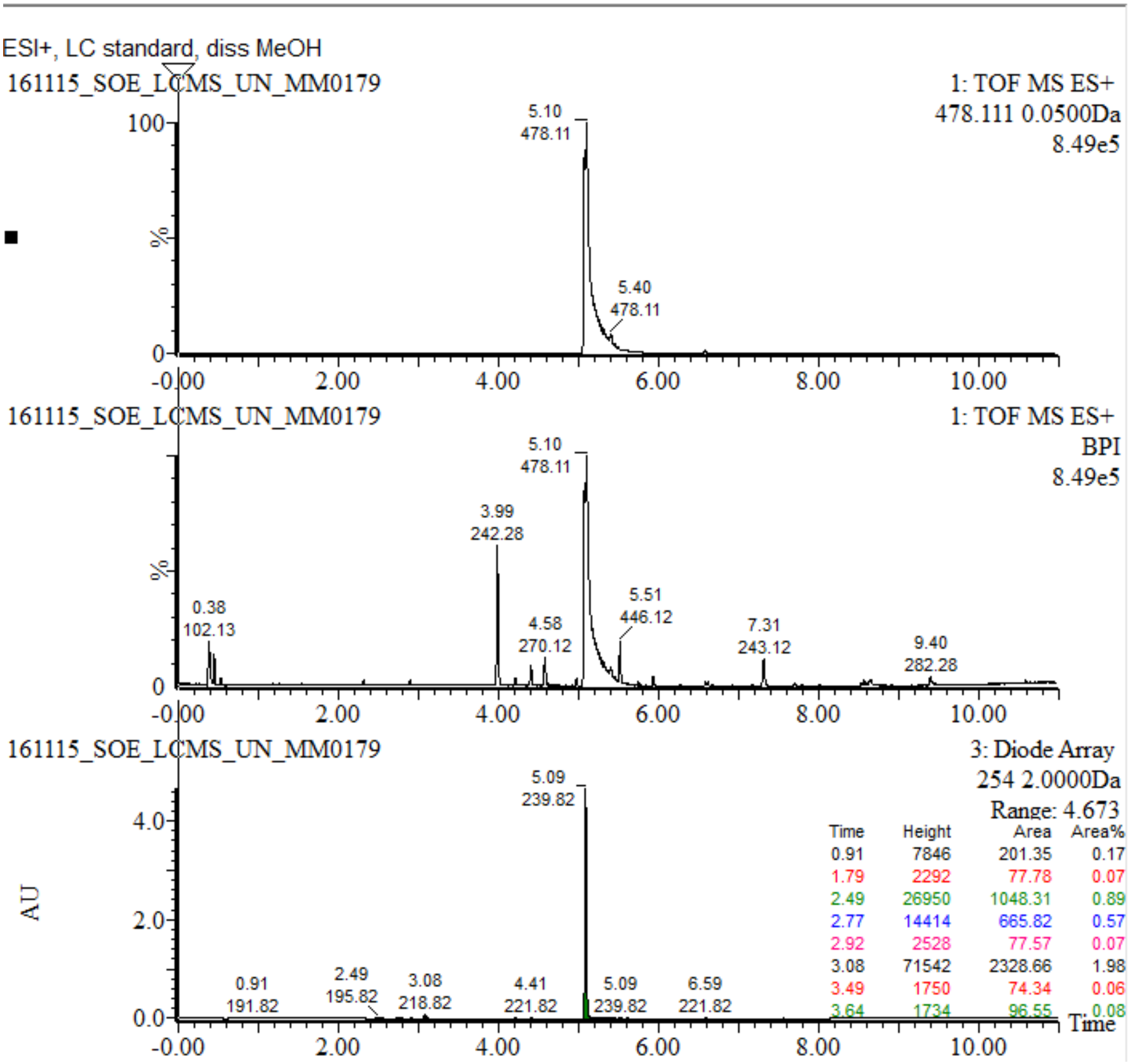
ESI+, LC standard, diss MeOH
161115_SOE_LCMS_UN_MM0179
1: TOF MS ES+
478.111 0.0500Da
8.49e5
5.10
478.11
5.40
478.11
100
%
0
-0.00
2.00
4.00
6.00
8.00
10.00
161115_SOE_LCMS_UN_MM0179
1: TOF MS ES+
BPI
8.49e5
5.10
478.11
3.99
242.28
0.38
102.13
4.58
270.12
5.51
446.12
7.31
243.12
9.40
282.28
161115_SOE_LCMS_UN_MM0179
3: Diode Array
254 2.0000Da
Range: 4.673
5.09
239.82
AU
4.0
2.0
0.0
0.91
191.82
2.49
195.82
3.08
218.82
4.41
221.82
5.09
239.82
6.59
221.82
Time Height Area Area%
0.91 7846 201.35 0.17
1.79 2292 77.78 0.07
2.49 26950 1048.31 0.89
2.77 14414 665.82 0.57
2.92 2528 77.57 0.07
3.08 71542 2328.66 1.98
3.49 1750 74.34 0.06
3.64 1734 96.55 0.08
Time

Single Mass Analysis
Tolerance = 3.0 mDa / DBE: min = -1.5, max = 100.0
Element prediction: Off
Number of isotope peaks used for i-FIT = 3
Monoisotopic Mass, Even Electron Ions
1841 formula(e) evaluated with 12 results within limits (up to 10 closest results for each mass)
Elements Used:
C: 0-100 H: 0-100 N: 0-8 O: 0-10 S: 1-2 F: 0-1

Mass	Calc. Mass	mDa	PPM	DBE	Formula	Fit Conf %	C	H	N	O	S	F
478.1105	478.1107	-0.2	-0.4	11.5	C21 H24 N3 O6 S2	36.48	21	24	3	6	2	
	478.1099	0.6	1.3	20.5	C30 H21 N S2 F	1.63	30	21	1		2	1
	478.1098	0.7	1.5	17.5	C22 H17 N7 O3 S F	3.61	22	17	7	3	1	1
	478.1113	-0.8	-1.7	20.5	C29 H20 N O4 S	0.04	29	20	1	4	1	
	478.1118	-1.3	-2.7	7.5	C18 H25 N3 O7 S2 F	39.59	18	25	3	7	2	1
	478.1120	-1.5	-3.1	16.5	C22 H20 N7 O2 S2	7.25	22	20	7	2	2	
	478.1086	1.9	4.0	21.5	C25 H16 N7 O2 S	0.24	25	16	7	2	1	
	478.1124	-1.9	-4.0	16.5	C26 H21 N O5 S F	0.15	26	21	1	5	1	1
	478.1084	2.1	4.4	12.5	C21 H21 N3 O7 S F	11.00	21	21	3	7	1	1
	478.1126	-2.1	-4.4	25.5	C30 H16 N5 S	0.01	30	16	5		1	

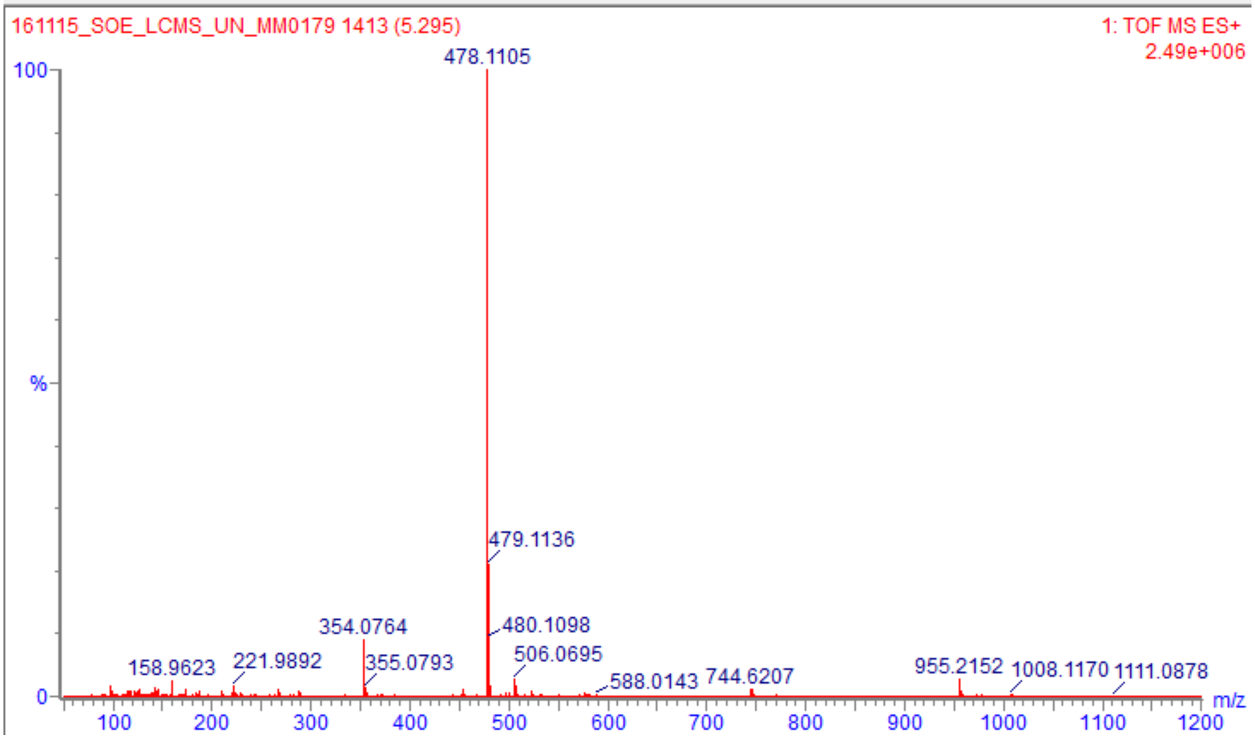

Compound: 6a

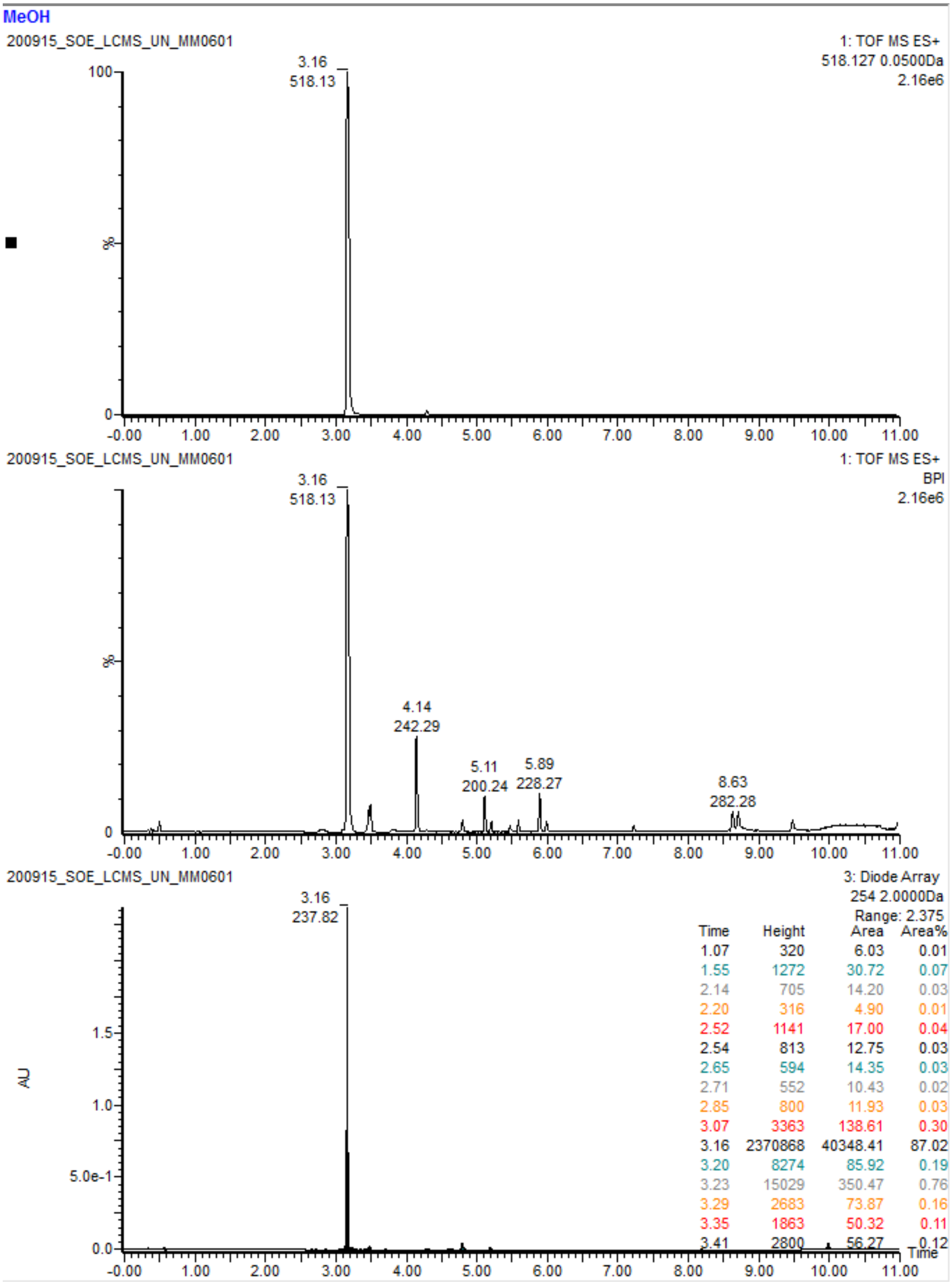
MeOH
200915_SOE_LCMS_UN_MM0601
1: TOF MS ES+
518.127 0.0500Da
2.16e6
3.16
518.13
100
%
0
-0.00 1.00 2.00 3.00 4.00 5.00 6.00 7.00 8.00 9.00 10.00 11.00
200915_SOE_LCMS_UN_MM0601
1: TOF MS ES+
BPI
2.16e6
3.16
518.13
4.14
242.29
5.11
200.24
5.89
228.27
8.63
282.28
%
0
-0.00 1.00 2.00 3.00 4.00 5.00 6.00 7.00 8.00 9.00 10.00 11.00
200915_SOE_LCMS_UN_MM0601
3: Diode Array
254 2.0000Da
Range: 2.375
3.16
237.82
AU
1.5
1.0
5.0e-1
0.0
Time Height Area Area%
1.07 320 6.03 0.01
1.55 1272 30.72 0.07
2.14 705 14.20 0.03
2.20 316 4.90 0.01
2.52 1141 17.00 0.04
2.54 813 12.75 0.03
2.65 594 14.35 0.03
2.71 552 10.43 0.02
2.85 800 11.93 0.03
3.07 3363 138.61 0.30
3.16 2370868 40348.41 87.02
3.20 8274 85.92 0.19
3.23 15029 350.47 0.76
3.29 2683 73.87 0.16
3.35 1863 50.32 0.11
3.41 2800 56.27 0.12
-0.00 1.00 2.00 3.00 4.00 5.00 6.00 7.00 8.00 9.00 10.00 11.00
Time

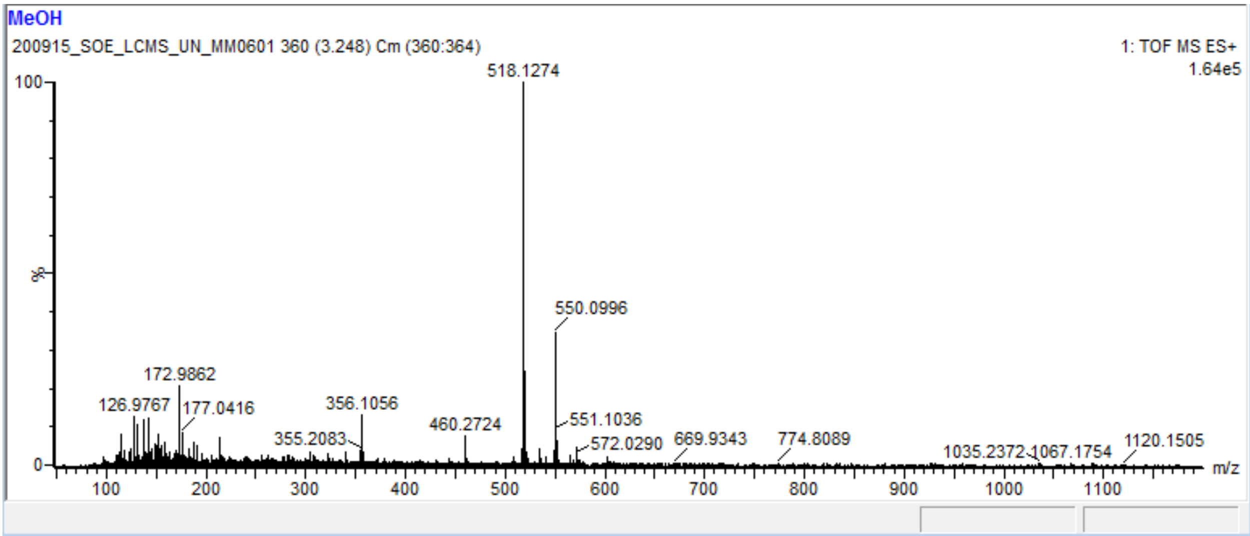

Single Mass Analysis
Tolerance = 2.0 mDa / DBE: min = -1.5, max = 50.0
Element prediction: Off
Number of isotope peaks used for i-FIT = 3
Monoisotopic Mass, Even Electron Ions
550 formula(e) evaluated with 2 results within limits (up to 10 closest results for each mass)
Elements Used:

Mass	Calc. Mass	mDa	PPM	DBE	Formula	i-FI...	Fit Conf %	C	H	N	O	S
518.1274	518.1273	0.1	0.2	17.5	C28 H24 N O7 S	34.880	0.76	28	24	1	7	1
	518.1267	0.7	1.4	8.5	C20 H28 N3 O9 S2	20.008	99.24	20	28	3	9	2

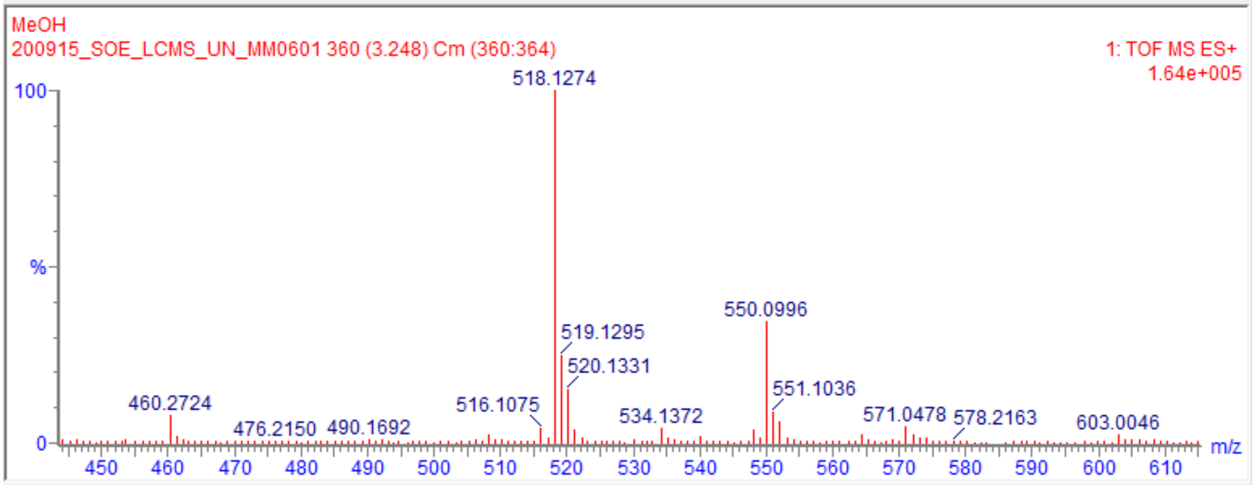

Compound: 6b

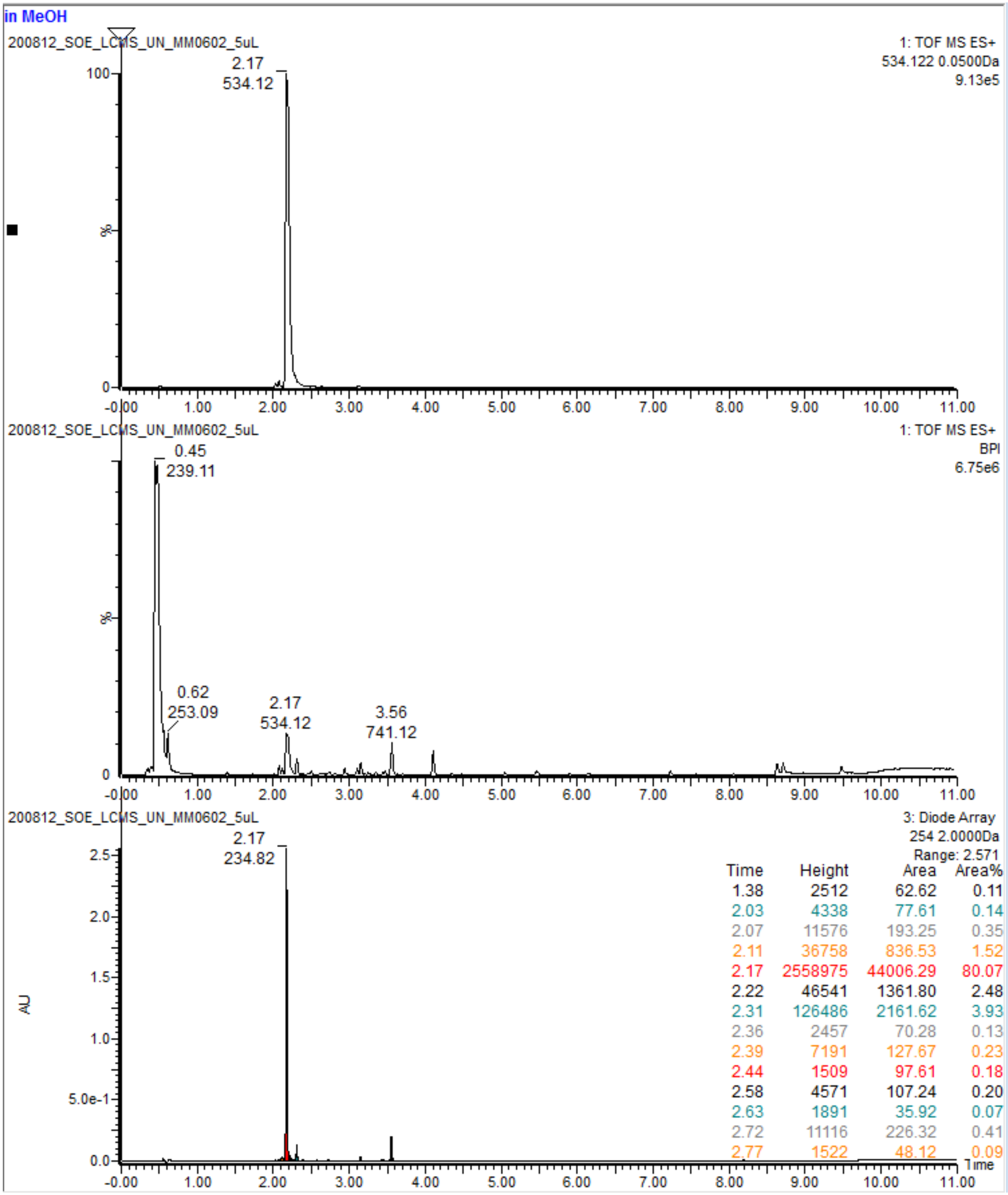

Time	Height	Area	Area%
1.38	2512	62.62	0.11
2.03	4338	77.61	0.14
2.07	11576	193.25	0.35
2.11	36758	836.53	1.52
2.17	2558975	44006.29	80.07
2.22	46541	1361.80	2.48
2.31	126486	2161.62	3.93
2.36	2457	70.28	0.13
2.39	7191	127.67	0.23
2.44	1509	97.61	0.18
2.58	4571	107.24	0.20
2.63	1891	35.92	0.07
2.72	11116	226.32	0.41
2.77	1522	48.12	0.09

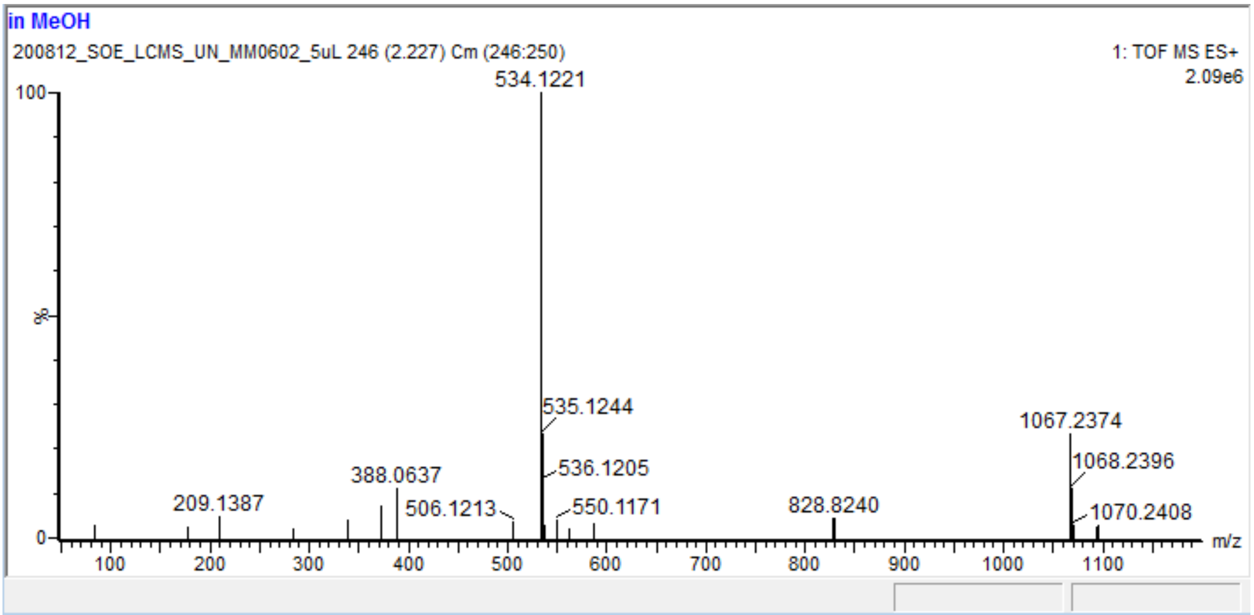

Single Mass Analysis
Tolerance = 2.0 mDa / DBE: min = -1.5, max = 50.0
Element prediction: Off
Number of isotope peaks used for i-FIT = 3
Monoisotopic Mass, Even Electron Ions
1130 formula(e) evaluated with 3 results within limits (up to 10 closest results for each mass)
Elements Used:
C: 0-80 H: 0-100 N: 0-6 O: 0-15 S: 1-2

Mass	Calc. Mass	mDa	PPM	DBE	Formula	Fit Conf %	C	H	N	O	S
534.1221	534.1223	-0.2	-0.4	17.5	C28 H24 N O8 S	310.00	28	24	1	8	1
	534.1216	0.5	0.9	8.5	C20 H28 N3 O10 S2	20100.00	20	28	3	10	2
	534.1236	-1.5	-2.8	22.5	C29 H20 N5 O4 S	310.00	29	20	5	4	1

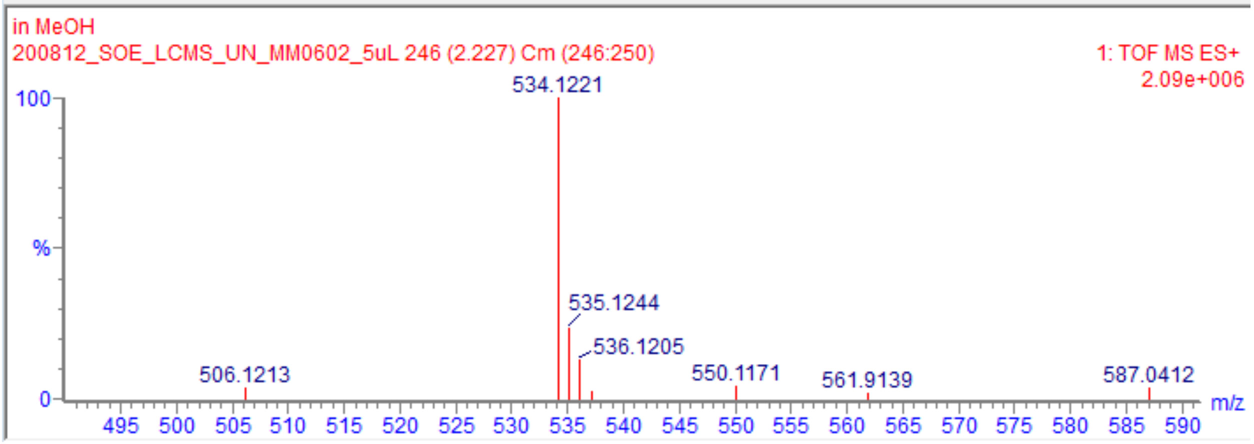

Compound: 6c

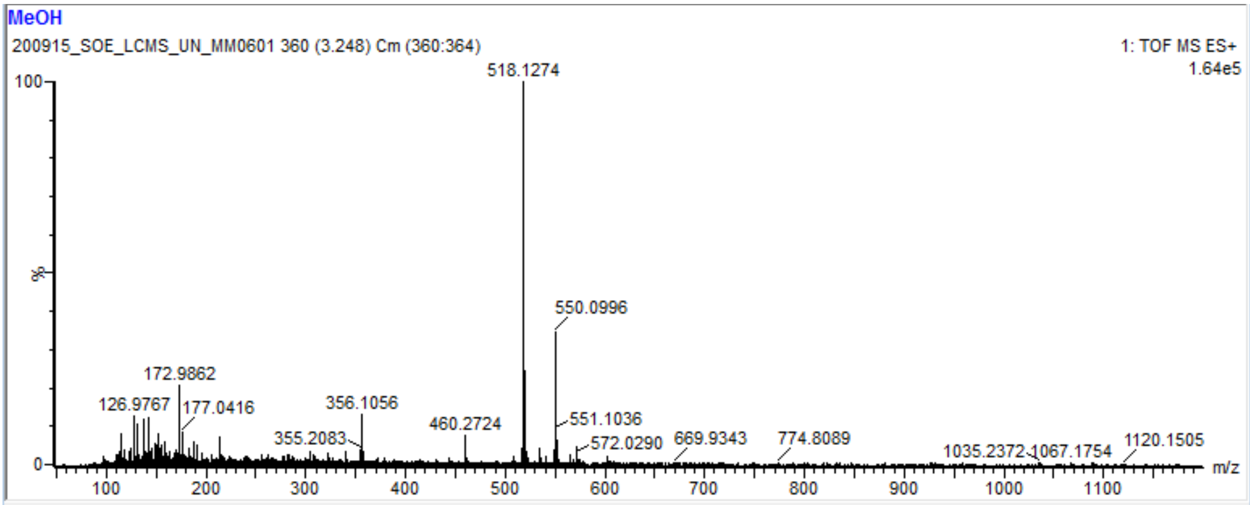

Single Mass Analysis
Tolerance = 2.0 mDa / DBE: min = -1.5, max = 50.0
Element prediction: Off
Number of isotope peaks used for i-FIT = 3
Monoisotopic Mass, Even Electron Ions
550 formula(e) evaluated with 2 results within limits (up to 10 closest results for each mass)
Elements Used:

Mass	Calc. Mass	mDa	PPM	DBE	Formula	i-FI...	Fit Conf %	C	H	N	O	S
518.1274	518.1273	0.1	0.2	17.5	C28 H24 N O7 S	34.880	0.76	28	24	1	7	1
	518.1267	0.7	1.4	8.5	C20 H28 N3 O9 S2	20.008	99.24	20	28	3	9	2

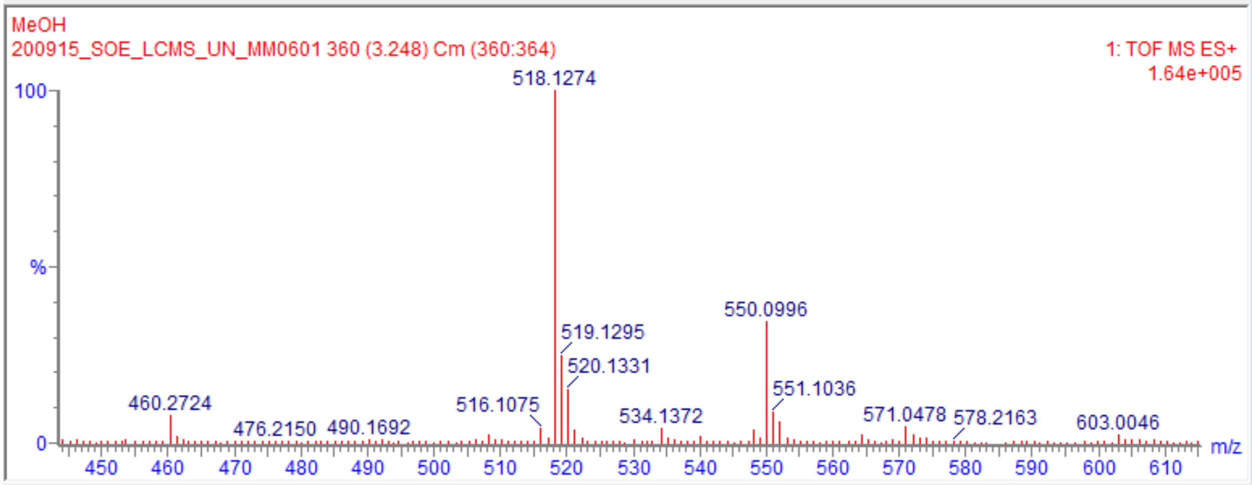

Data File D:\Data\MM\gal-glu-sulfone 2020-06-22 21-21-52.D
Sample Name: gal-glu-sulfone MM603

6c

===

Acq. Operator : SYSTEM
Sample Operator : SYSTEM
Acq. Instrument : LCMS Location : 1
Injection Date : 6/22/2020 9:23:01 PM Inj : 1
Inj Volume : 5.000 µl
Acq. Method : C:\Chem32\1\Methods\Standard_LC_5min.M
Last changed : 6/22/2020 9:19:03 PM by SYSTEM
(modified after loading)
Analysis Method : C:\Chem32\1\Methods\Standard_LC_5min.M
Last changed : 3/31/2021 8:46:44 AM by SYSTEM

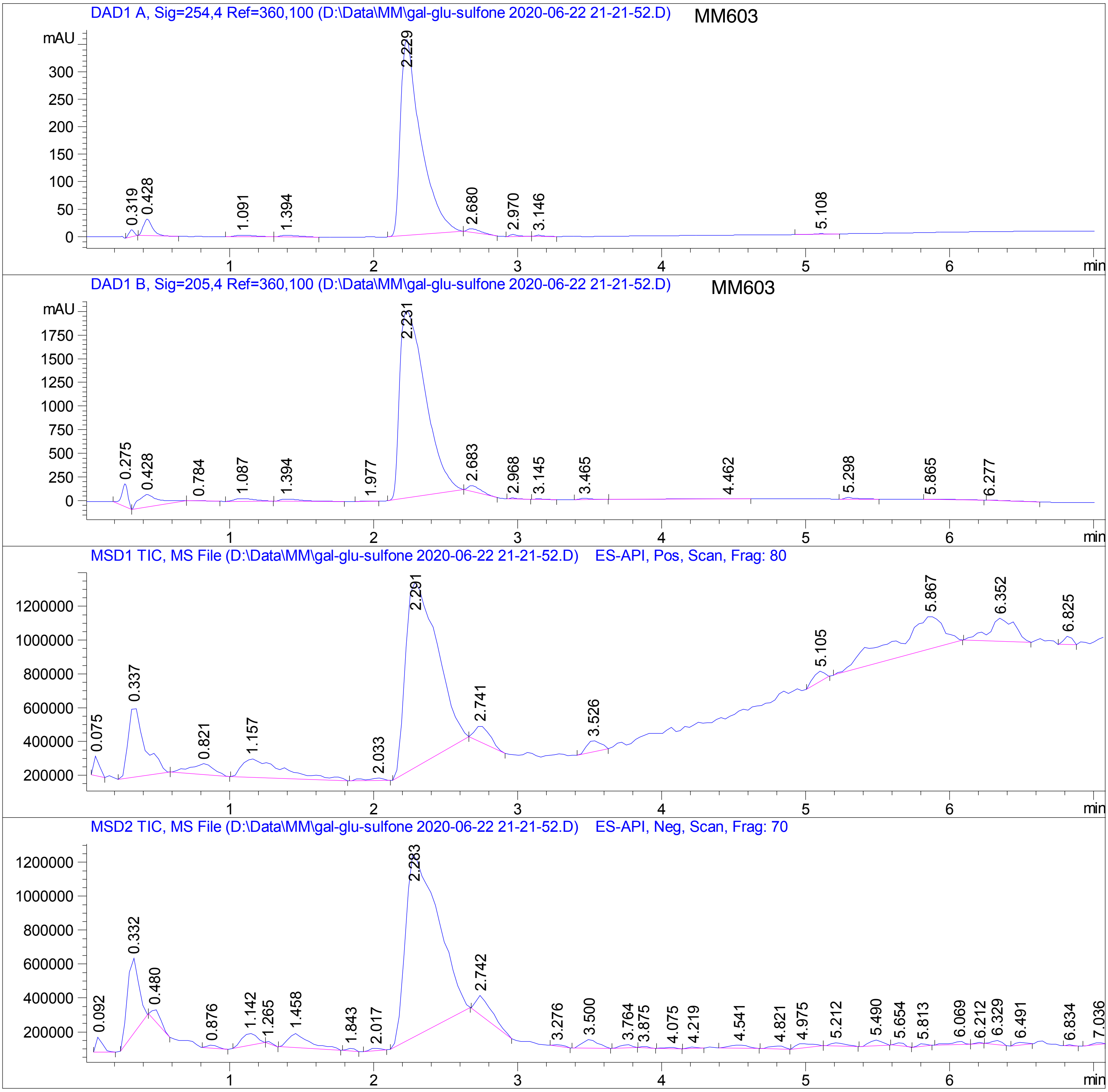

6c

Area Percent Report

Sorted By : Signal
Multiplier : 1.0000
Dilution : 1.0000
Sample Amount: : 1.00000 [ng/ul] (not used in calc.)
Use Multiplier & Dilution Factor with ISTDs

Signal 1: DAD1 A, Sig=254,4 Ref=360,100

Peak #	RetTime [min]	Type	Width [min]	Area [mAU*s]	Height [mAU]	Area %
1	0.319	BB	0.0377	30.26690	12.73572	0.7985
2	0.428	BB	0.0668	129.69952	29.79985	3.4219
3	1.091	BB	0.1525	29.69542	3.08001	0.7835
4	1.394	BB	0.1482	24.94203	2.63979	0.6581
5	2.229	BB	0.1404	3502.81030	357.32452	92.4166
6	2.680	BB	0.0953	42.65741	6.60942	1.1255
7	2.970	BB	0.0568	14.37847	3.74190	0.3794
8	3.146	BB	0.0535	8.82149	2.48279	0.2327
9	5.108	BB	0.0904	6.96604	1.06312	0.1838
Totals :				3790.23758	419.47713	

Signal 2: DAD1 B, Sig=205,4 Ref=360,100

Peak #	RetTime [min]	Type	Width [min]	Area [mAU*s]	Height [mAU]	Area %
1	0.275	BB	0.0462	710.65350	242.28297	2.5603
2	0.428	BB	0.1300	1291.39221	132.14377	4.6525
3	0.784	BB	0.0929	27.53093	4.66164	0.0992
4	1.087	BB	0.1518	273.63300	28.56384	0.9858
5	1.394	BB	0.1587	227.87892	22.39487	0.8210
6	1.977	BB	0.0895	10.99428	1.95829	0.0396
7	2.231	BB	0.1726	2.44620e4	1974.00256	88.1301
8	2.683	BB	0.0913	387.80969	61.76474	1.3972
9	2.968	BB	0.0528	35.87151	10.24728	0.1292
10	3.145	BB	0.0532	32.30448	9.15823	0.1164
11	3.465	BB	0.0709	71.31155	14.61542	0.2569
12	4.462	BB	0.4373	83.13627	2.28040	0.2995
13	5.298	BB	0.0703	89.17159	17.84352	0.3213
14	5.865	BB	0.2389	39.78214	2.04567	0.1433
15	6.277	BB	0.1460	13.21784	1.16814	0.0476
Totals :				2.77567e4	2525.13135	

6c

Signal 3: MSD1 TIC, MS File

Peak #	RetTime [min]	Type	Width [min]	Area	Height	Area %
1	0.075	BB	0.0454	3.20045e5	1.17612e5	1.0058
2	0.337	BB	0.1113	3.34204e6	4.21229e5	10.5026
3	0.821	BB	0.1625	7.36311e5	6.39035e4	2.3139
4	1.157	BB	0.2813	2.31126e6	1.10576e5	7.2633
5	2.033	BB	0.1184	1.25925e5	1.47400e4	0.3957
6	2.291	BB	0.2151	1.66737e7	1.09508e6	52.3983
7	2.741	BB	0.1252	7.18359e5	9.56576e4	2.2575
8	3.526	BB	0.1094	4.64747e5	6.67179e4	1.4605
9	5.105	BB	0.1078	3.49120e5	5.99818e4	1.0971
10	5.867	BB	0.3341	4.86241e6	1.91255e5	15.2805
11	6.352	BB	0.1771	1.71988e6	1.35534e5	5.4049
12	6.825	BB	0.0654	1.97219e5	5.02626e4	0.6198
Totals :				3.18210e7	2.42255e6	

Signal 4: MSD2 TIC, MS File

Peak #	RetTime [min]	Type	Width [min]	Area	Height	Area %
1	0.092	BB	0.0510	2.72889e5	8.91668e4	1.1152
2	0.332	BB	0.0930	2.48954e6	4.47319e5	10.1735
3	0.480	BB	0.0781	2.93672e5	6.86111e4	1.2001
4	0.876	BB	0.0840	8.91575e4	1.76988e4	0.3643
5	1.142	BB	0.1234	4.99995e5	6.78818e4	2.0432
6	1.265	BB	0.0565	3.25533e4	9607.22949	0.1330
7	1.458	BB	0.1507	8.89916e5	8.47067e4	3.6366
8	1.843	BB	0.0613	5.75991e4	1.56609e4	0.2354
9	2.017	BB	0.0901	7.19357e4	1.40345e4	0.2940
10	2.283	BB	0.2080	1.68618e7	1.09342e6	68.9054
11	2.742	BB	0.1205	9.04759e5	1.14243e5	3.6973
12	3.276	BB	0.0942	5.22674e4	9252.18848	0.2136
13	3.500	BB	0.1234	3.77287e5	5.09444e4	1.5418
14	3.764	BB	0.0912	9.59806e4	1.77462e4	0.3922
15	3.875	BB	0.0640	2.70357e4	7037.30859	0.1105
16	4.075	BB	0.0753	3.52834e4	7277.03369	0.1442
17	4.219	BB	0.0828	3.82832e4	8171.16797	0.1564
18	4.541	BB	0.1464	1.73513e5	1.85891e4	0.7091
19	4.821	BB	0.1163	1.23190e5	1.82398e4	0.5034
20	4.975	BB	0.1220	2.06464e5	2.56441e4	0.8437
21	5.212	BB	0.1242	1.43629e5	1.93349e4	0.5869
22	5.490	BB	0.1101	2.05415e5	3.30231e4	0.8394
23	5.654	BB	0.0702	7.52964e4	1.78831e4	0.3077
24	5.813	BB	0.0615	5.26952e4	1.42898e4	0.2153
25	6.069	BB	0.0988	1.22024e5	1.77893e4	0.4987
26	6.212	BB	0.0445	1.51910e4	5685.35596	0.0621
27	6.329	BB	0.0746	1.12811e5	2.51881e4	0.4610
28	6.491	BB	0.0798	8.10991e4	1.54848e4	0.3314
29	6.834	BB	0.0426	1.61361e4	6316.43262	0.0659

Peak #	RetTime [min]	Type	Width [min]	Area	Height	Area %
30	7.036	BBA	0.0761	5.35120e4	1.17187e4	0.2187

Totals :	2.44709e7	2.35196e6

*** End of Report ***
